# Wireless Power Transfer Techniques for Implantable Medical Devices: A Review

**DOI:** 10.3390/s20123487

**Published:** 2020-06-19

**Authors:** Sadeque Reza Khan, Sumanth Kumar Pavuluri, Gerard Cummins, Marc P. Y. Desmulliez

**Affiliations:** 1 Institute of Sensors, Signals, and Systems, School of Engineering and Physical Sciences, Heriot-Watt University, Edinburgh EH14 4AS, UK; sumanth_kumar.pavuluri@hw.ac.uk (S.K.P.); m.desmulliez@hw.ac.uk (M.P.Y.D.); 2 School of Engineering, University of Birmingham, Birmingham B15 2TT, UK; g.cummins@bham.ac.uk

**Keywords:** acoustic coupling, capacitive coupling, electromagnetic, implantable medical device, optical power transfer, tissue safety, wireless power transfer

## Abstract

Wireless power transfer (WPT) systems have become increasingly suitable solutions for the electrical powering of advanced multifunctional micro-electronic devices such as those found in current biomedical implants. The design and implementation of high power transfer efficiency WPT systems are, however, challenging. The size of the WPT system, the separation distance between the outside environment and location of the implanted medical device inside the body, the operating frequency and tissue safety due to power dissipation are key parameters to consider in the design of WPT systems. This article provides a systematic review of the wide range of WPT systems that have been investigated over the last two decades to improve overall system performance. The various strategies implemented to transfer wireless power in implantable medical devices (IMDs) were reviewed, which includes capacitive coupling, inductive coupling, magnetic resonance coupling and, more recently, acoustic and optical powering methods. The strengths and limitations of all these techniques are benchmarked against each other and particular emphasis is placed on comparing the implanted receiver size, the WPT distance, power transfer efficiency and tissue safety presented by the resulting systems. Necessary improvements and trends of each WPT techniques are also indicated per specific IMD.

## 1. Introduction

Wireless power transfer (WPT) can be defined as a technology capable of transmitting energy across a medium, from a power source to an electrical load, without the use of electrical wires connecting this power source to the load [1,2]. This technology is extensively used in a wide range of applications ranging from sophisticated low-power biomedical implants [3,4,5,6], to high-power electric vehicles [7,8,9,10] to white goods such as electric toothbrushes and mobile phones [11].

**Figure 1:**
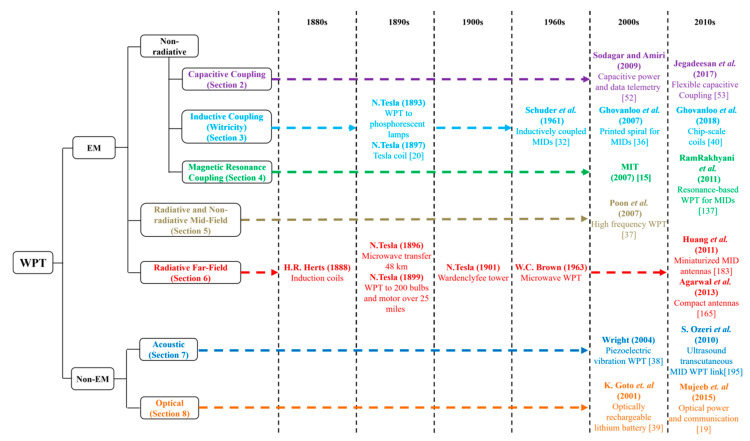
Classification and research overview of wireless power transfer (WPT) techniques indicating the key milestones relevant to implantable medical devices (IMDs).

Taxonomy of WPT techniques is provided in Figure 1 alongside these key technology breakthroughs from the 1880s to the present day, which are relevant to implantable medical devices (IMDs).These techniques use both electromagnetic (EM) and non-EM energy. The former includes electric, magnetic and optical coupling systems, which can be further classified as non-radiative transfer or as near-field systems, i.e., less than 100 mm distance between transmitter and IMD [12], and radiative transfer systems. Midfield WPT, characterized by a separation distance of 100 to 500 mm, lies in between the non-radiative and radiative regions [13]. Furthermore, non-radiative techniques include inductive coupling, which includes witricity [14], magnetic resonance coupling [15] and capacitive coupling [16]. Witricity, a term coined for wireless electricity, is the technology to transfer power using two resonating objects synchronized at the same operating frequency [17].The predominant non-EM technique is acoustic-based power transfer [18] and is a far-field (more than 500 mm distance) transmission technique similar to optical coupling [19].

One of the first demonstrations of WPT was carried out by Nikola Tesla in 1889 through the use of magnetic resonance and near-field coupling-based wireless power transfer coils, also known as Tesla coils [20]. Electromagnetic radiation was previously confirmed 10years earlier in 1888 by H. R. Hertz, when he successfully transmitted electricity over a tiny gap using induction coils. Used on the 1970s for high-frequency heating equipment [21,22], WPT systems were applied for short-range applications, such as inductive power transfer systems in the 1990s [21,22,23,24,25] and wireless charging systems for portable equipment in the 2000s [26,27,28,29,30]. Presently, WPT systems are commercially available for some applications while still being developed and improved for others, as explained later.

Miniaturized, multi-functional IMDs are growing in importance as they enable continuous monitoring, early-stage detection and initial treatment of dysfunctional organs. The power requirement associated with some of these devices is, however, a significant challenge. Transdermal or percutaneous wiring is inconvenient because of the large size of these wiring systems and their susceptibility to infect surrounding tissues. For deeper IMDs, a transcutaneous wire-based power source is a medically unacceptable solution as it leads to significant scarring of the tissue surrounding the IMD. Packaged batteries are limited by their operational lifetime [31], necessitating repeated surgical interventions to replace them. Additionally, any leakage from the battery could pose a severe risk to the patient’s health. Batteries also occupy a significant portion of the available internal volume of these miniaturized IMDs, which could reduce the functionality of such devices. Therefore, power requirement, longevity and size of the power supply dictate today’s specifications of modern IMDs.

WPT is potentially capable of fulfilling these requirements and enabling seamless and safe operation of IMDs. Over the last two decades, different WPT techniques targeted for IMDs, as shown in Figure 1, have been studied to improve the performance in terms of power transfer efficiency (PTE), system size and tissue safety. The application of WPT for medical implants started in the 1960s, through the work of Shuder et al., who used inductive coupling to power an artificial heart [32,33,34]. One of the earliest reviews of such technologies can be found here [35]. Significant advances occurred in the 2000s and 2010s. Research on printed spiral coils (PSCs), manufacturing techniques and performance characterization intensified from 2007 onwards [36]. This type of WPT coils is suitable for IMDs as it can be printed and mounted conveniently. Magnetic resonance coupling for IMDs attracted the attention of researchers since its invention in 2007 at MIT [15]. Research on mid-field [37], acoustic piezoelectric vibration-based [38] and optical [39] WPT techniques in the 2000s have also introduced the possibility of miniaturized WPT RX coils for deep implants. Recently invented chip-scale WPT coils [40] provided further miniaturization potential for IMDs.

Multiple research studies have been published over the last few years addressing the technological development of WPT techniques. Most of these publications focused on a single aspect of WPT. Complete overviews on WPT methods and their relevant potential application for MIDs are rare.

In [41], a generalized and fundamental overview of WPT was presented. Furthermore, the authors in [42,43] described the theory behind mid-field WPT and its recent improvements. In [44], the challenges, possible design procedures and future of far-field WPT were examined. The possibility of using metamaterials for WPT was reviewed in [45,46]. Radio frequency (RF) modeling, different frequency bands and a brief study on the RF implantable device testing inside phantoms, ex vivo and in vivo were presented in [47]. The mid-field WPT for implantable systems was analyzed in [48] and its powering method for cardiac implants was demonstrated. In [49,50], the possibility and few applications of WPT for capsule endoscopy were demonstrated. Furthermore, the acoustic WPT technique for MIDs was reviewed in [18]. Recently, in [12,51], the theoretical aspects of non-radiative near-field WPT were examined in details. These articles mainly cover the inductive coupling link for WPT. In [35], different WPT techniques and their design issues are reviewed. This work focused on the neural implant application of WPT. Few other applications based on inductively coupled WPT are presented. However, this work completely leaves out the applications of magnetic resonance coupling and far-field WPT. Only one application of mid-field and acoustic power transfer is presented in this article. The article also overlooks the possibility of optical power transfer for MIDs. Therefore, a complete guide of different WPT techniques and their potential MID applications implemented by different research groups is absent in the literature.

In this paper, a systematic review based on the system design and optimization methodology, performance and implementation of currently available WPT technology for IMDs are presented. Section 2, Section 3, Section 4, Section 5, Section 6, Section 7 and Section 8 discuss the various WPT methods proposed for powering IMDs following the taxonomy shown in Figure 1. These sections describe the theoretical aspects and operation principles of the WPT links presented in this paper. The analytical design and optimization method of these WPT techniques are explained briefly alongside the expression of the power transfer efficiency (PTE). PTE is adopted as the primary evaluation factor in this review as it is the most significant parameter for evaluating and comparing the performance of a WPT link for any application.

For each WPT technique, the respective transmitter and receiver circuits are presented alongside the electronic circuitry used for conditioning the power received. Power transfer efficiency values for a specific WPT link (i.e., the efficiency of the system without the electronic circuit) are included whenever it is available in the literature. This is followed by a presentation of the range of applications using this WPT technique. Finally, a presentation of the design challenges and future design trends is provided, followed by the advantages and drawbacks of each technique.

Section 2 describes the capacitively coupled WPT system with Section 3 and Section 4, presenting in detail the well-established non-radiative inductively coupled and magnetic resonance based WPT techniques. Section 5 and Section 6 explain the more recent mid and far-field WPT methods. Section 7 summaries the potential of non-EM acoustic WPT technique. Section 8 includes the least studied non-EM optical WPT method. Finally, a brief comparison of these methods is conducted and presented in Section 9 followed by conclusions.

## 2. Non-Radiative Capacitor Coupling

### 2.1. Link Design

**Figure 2:**
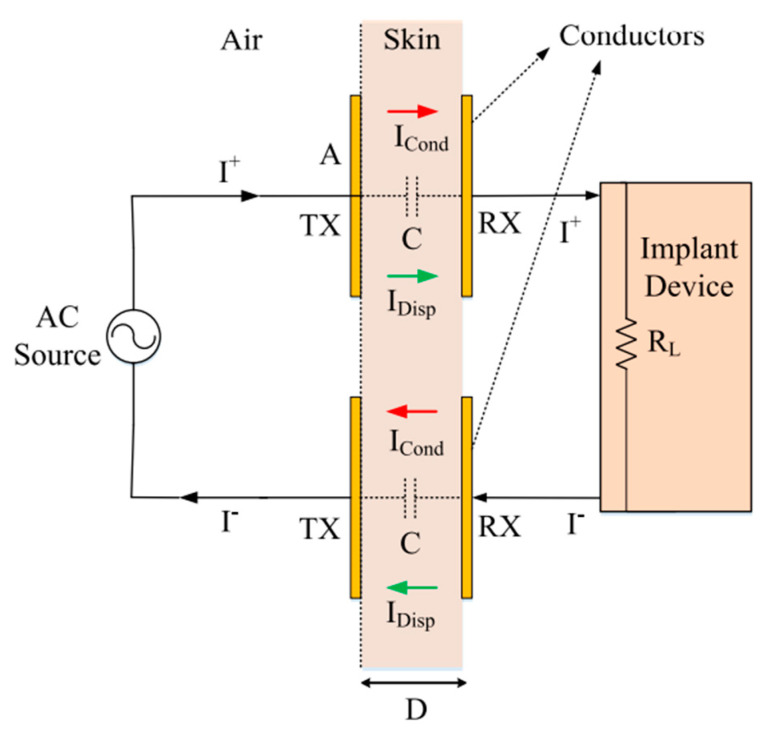
Non-radiative capacitive coupling (NRCC) method schematic (taken from [55]. Copyright ©2017, IEEE).

Non-radiative capacitive coupling (NRCC) of IMDs was first demonstrated for the powering of subcutaneous implants in [52] and was later extended to flexible implants. The coupling system is schematically depicted in Figure 2, which shows a pair of conductors placed on each side of the skin and separated by a distance *D* and connected to the implant device with a load resistance *R_L_* [36]. Another pair of conductors ensures a closed current loop whereby power can be delivered to the IMD. WPT across the tissue layers is established by the displacement current, *I_Disp_*, between the conductor plates. The voltage excitation on both transmitter/receiver (TX/RX) pairs generates extremely low currents due to the small effective area and therefore the small capacitance of the conductors. NRCC was initially investigated for wideband data telemetry [52,53,54] using digital modulation techniques, such as frequency shift keying (FSK) or binary phase-shift keying (BPSK). However, the wireless powering potential of NRCC has only been recently studied seriously.

### 2.2. Optimization

Optimization of the WPT link relies on satisfying several trade-offs. The conduction current, *I_cond_*, induced in the skin and surrounding tissues, must be minimized to reduce dispersive losses and EM field decay rate [56]. This can be implemented either by increasing *D*, which favors deep implantation of the medical device or operating at low frequencies due to the increase in conductivity with decreasing frequency. On the other hand, the displacement current must be maximized for optimum PTE, which requires either a decrease of *D* or increase of the effective area, *A*, of the conductors, which limit the application range of IMDs, an increase of frequency to reduce the complex, frequency-dependent relative dielectric permittivity, *ε_r_*(*ω*), or an increased rate of change of the electric field.

The optimized PTE, *η*, can be approximated using the dielectric tissue dispersion of the Cole-Cole relaxation model [55,57]:

(1) $$\eta =\frac{R_{L}}{R_{L}+R_{T}}\left(1-{\left|\tau \right|}^{2}\right)$$

(2) $$R_{T}=Real\left(\frac{-jD}{\omega \epsilon_{o}\varepsilon_{r}\left(\omega \right)A}\right)$$

where *R_T_* describes the loss resistance equivalent to tissue losses, *τ* is the reflection coefficient, and *ϵ_o_* is the free space permittivity. The calculation of *R_T_* assumes that the electric fields are localized within the volume defined between the TX and RX patches. Depending on the nature of the tissues between the conductors, NRCC requires an operating frequency of tens of MHz to achieve good power transfer efficiency. Higher frequencies would increase conduction losses in the tissues and, close to the tissue relaxation resonance (in the GHz range), would cause severe damage [58].

### 2.3. System Design

**Figure 3:**
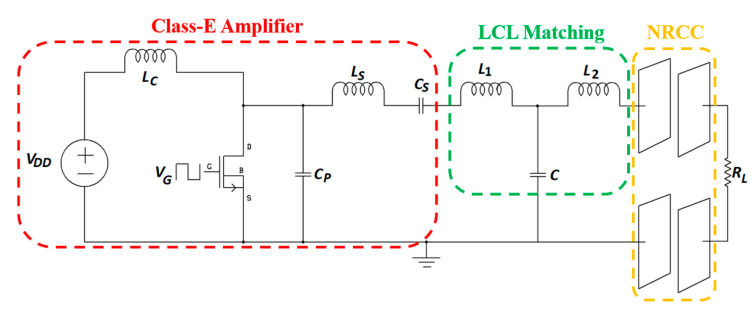
NRCC WPT system [59].

A description of NRCC-specific RX power circuitry, including rectifier and regulator, is rare in the literature. In [59], a nominal condition-based class-E power amplifier (PA) is proposed for NRCC presenting a load resistance *R_L_*, as shown in Figure 3.

The class-E power amplifier (PA) is designed with a CSD13380F3 N-channel MOSFET switch with a TPS28226 high-frequency driver. *L_c_* is the radio frequency choke (RFC) inductor to provide a constant current to the circuit. The optimized shunt capacitance, *C_P_*, the resonant circuit inductance, *L_S_*, and the capacitance, *C_S_*, are given as:

(3) $$C_{P}=\frac{\left(\begin{array}{c} P_{o}\left(1-\delta \right)\cos\left(2\pi \delta +\varphi \right)[\pi \left(1-\delta \right)\\ \cos\left(2\pi \delta \right)+\sin\left(2\pi \delta \right)] \end{array}\right)}{\omega V_{dc}^{2}\sin\left(2\pi \delta +\varphi \right)\sin\left(2\pi \delta \right)}$$

(4) $$L_{S}=\frac{V_{DD}^{2}}{2\pi \omega P_{o}}\left\{\begin{array}{c} 2\pi^{2}\delta^{2}-2\pi \delta V_{Z}\cos\left(\varphi \right)+V_{Z}\\ \left[\sin\left(2\pi \delta +\varphi \right)-\sin\varphi \right] \end{array}\right\}$$

(5) $$C_{S}=\frac{1}{\omega L_{S}}$$

where *P_o_* is the output power, *δ* is the duty ratio, *φ* is the initial phase shift of the load current and *V_DD_* is the dc voltage source. An inductance-capacitance-inductance (LCL) impedance matching network (*L*_1_, *L*_2_ and *C*) is also used to improve the PA efficiency under the variable load condition. In [59], a maximum PA efficiency of 96.34% was claimed for 13.56 MHz operating frequency. The primary design concern of the proposed system is the power consumption of the TPS28226 driver at higher frequencies. According to the technical datasheet, this particular driver can dissipate approximately 1.1 W of power for a switching frequency of less than 2 MHz. Therefore, the power dissipation would be very high for an operating frequency of 13.56 MHz. It is unclear whether the dissipated power by the driver has been included in the final PA efficiency calculation.

### 2.4. Applications

NRCC studies as a WPT scheme for biomedical applications are still at an early stage. In [53], an NRCC WPT link was tested on a non-human primate cadaver between 100 to 150 MHz for a 40 × 40 mm^2^ external TX patch size. For a 20 × 20 mm^2^ RX patch, the PTE was calculated as 56% for a separation distance of 7 mm. The maximum specific absorption rate (SAR) was 8.02 W/kg for 1 W of input power. It is a measure of the rate at which energy is absorbed per unit mass by a human body when exposed to a radio frequency. The value of SAR must be lower than the IEEE standard of 2 W/kg for 10 g of tissue [60].

### 2.5. Design Challenges and Future Trends

NRCC has several major design challenges:

(1) The limited amount of power delivered by NRCC is an issue due to the low PTE. To improve the PTE, the capacitance must increase, requiring that the separation distance is very small, of the order of millimeters or below.
(2) NRCC is sensitive to misalignment. Any misalignment reduces the capacitance coupling and leads to a substantial decrease in the PTE.
(3) Efficient power processing (rectification) is difficult for a high operation frequency due to the difficulty in designing and implementing a high-efficiency rectifier for NRCC.
(4) Extensive tissue safety analysis should be demonstrated before considering this method for in vivo applications, as no report is yet available regarding tissue safety.

Accurate determination of the dielectric properties of the application-specific biological tissues would be essential to improve the PTE of the NRCC link. Further study and modeling of the effect of misalignment on the PTE is also an important topic of research for further improving NRCC WPT.

### 2.6. Verdict

NRCC WPT technique for IMDs is in an early stage of research. The PTE of such a technique depends on the size and distance between parallel plates. However, the larger size of the receiver coil is inappropriate for the RX side of the IMDs. It is possible to improve the PTE by increasing the operating frequency, which can lead to severe tissue damage situation. An improved optimization methodology is necessary for this technique representing the PTE as a function of capacitor plate size, distance and operation frequency. There is also a lack of adequate analysis of tissue safety associated with this technique. Furthermore, examples of the implementation of such a technique associated with in vivo testing are rare in the literature.

## 3. Non-Radiative Inductive Coupling

### 3.1. Link Design

Non-radiative inductive coupling (NRIC) is the oldest and most established WPT method and works according to the electromagnetic (EM) induction principle [36]. It is also known as loosely coupled WPT because of the separation distance is comparable with the size of the receiver coil. Several inductively coupled wireless implantable devices, such as cochlear implants and neurostimulators, have already been approved by the U.S Food and Drug Administration (FDA) [61,62].

**Figure 4:**
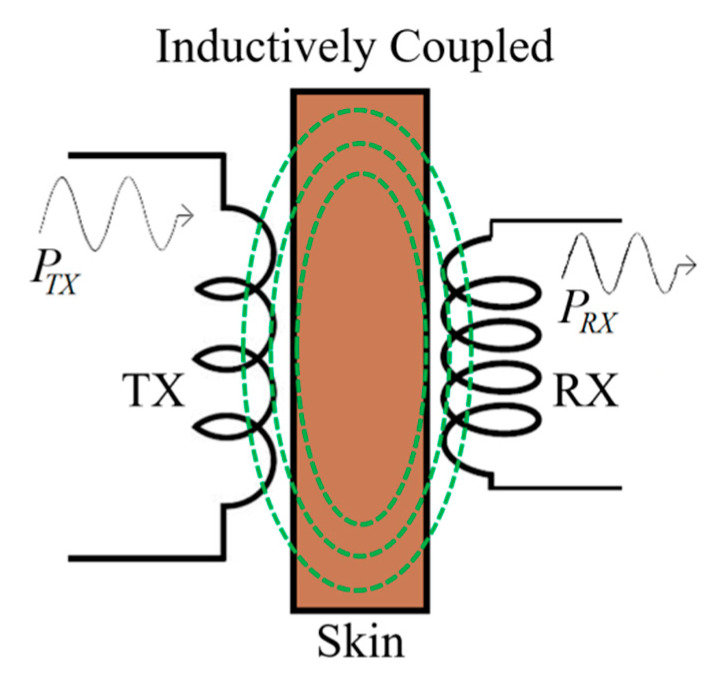
NRIC WPT system powered by alternative electromotive force (EMF). TX: transmitter coil RX; receiving coil.

As shown in Figure 4, a transmitter (TX) coil is positioned adjacent to the skin and supplies a time varying magnetic field generated by a high-frequency voltage driver source. This magnetic field induces an electromotive force (EMF) in the receiving (RX) coil placed inside the body, which is processed using a silicon-based rectifier with the RX system [51]. The RX coil should be tuned to the same operating frequency as the TX coil to increase the PTE [63].

### 3.2. Optimization

Several coil designs have been reported over the last two decades and range from wire wound coils (WWCs) [64,65] to PSCs [36,66,67,68]. The methodology to optimize the whole WPT scheme is, however, relatively similar for most of the reported systems. As this WPT scheme is predominantly used, a detailed description of the optimization method is provided here alongside the description of the main system components.

#### 3.2.1. Self-Inductance

The expression of the self-inductance, *L*, of the PSC used in most of the literature is [36,66,69,70,71,72]:

(6) $$L=\frac{C_{1}\mu N_{l}^{2}D_{avg}}{2}\left[\ln\left(\frac{C_{2}}{\varphi }+C_{3}\varphi +C_{4}\varphi^{2}\right)\right]$$

where *N_l_* is the number of loops, *µ* = *µ*_o_*µ_r_* is permeability and *D_avg_* = (*D_o_* + *D_i_*)/2, where *D_o_* and *D_i_* are the outer and inner diameters of the coil, respectively, and *φ* is the fill factor. The coefficients *C_i_* are layout-dependent as shown in Table 1 [73].

The analytical expression of the self-inductance, *L*, of a one-turn WWC is either [65,74,75,76]:

(7) $$L=\frac{\mu D_{o}}{2}\ln\left(\frac{D_{o}}{w}\right)$$

where *w* is the wire diameter of the loop, or [64,77,78]:

(8) $$L=\frac{\mu D_{o}}{2}\left(\ln\left(\frac{8D_{o}}{w}\right)-2\right)$$

For a multi-loop, multi-layer WWC, the self-inductance is the combination of the self-inductances of all the loops and layers and the mutual inductances among different loops and layers [77,78]. Additionally, an accurate estimation of the radius and length of the coil, inspired from the Archimedean spiral found in nature, is presented in [79] to improve the optimization performance.

#### 3.2.2. Mutual Inductance

A simplified form of the Maxwell’s equation is used in the estimation of the mutual inductance between two coils *C_i_* and *C_j_* of radii *R_i_* and *R_j_*. Used for PSCs [36,66,69,70,71] and WWCs [64,77], the expression is:

(9) $$M_{ij}=\frac{2\mu }{\alpha_{ij}}\sqrt{R_{i}R_{j}}\left[\left(1-\frac{\alpha_{ij}^{2}}{2}\right)K\left(\alpha_{ij}\right)-E\left(\alpha_{ij}\right)\right]$$

(10) $$\alpha_{ij}=2\sqrt{\frac{R_{i}R_{j}}{{\left(R_{i}+R_{j}\right)}^{2}+{d_{ij}}^{2}}}$$

where *d_ij_* is the distance between C*_i_* and C*_j_*, assumed perfectly aligned, and *K*(*α*) and *E*(*α*) are the complete elliptic integrals of the first and second kind, respectively. Another approach to calculate *M_ij_* stems from the approximation of the Neumann’s equation [80]:

(11) $$M_{ij}=\begin{array}{c} \frac{\mu \pi R_{i}^{2}R_{j}^{2}}{2{\left(R_{i}^{2}+R_{j}^{2}+d_{ij}^{2}\right)}^{\frac{3}{2}}}\left[1+\frac{15}{32}\gamma^{2}+\frac{315}{1024}\gamma^{4}\right] \end{array}$$

(12) $$\gamma =\frac{2R_{i}R_{j}}{R_{i}^{2}+R_{j}^{2}+d_{ij}^{2}}$$

The latter approach has the advantage to accommodate translational and angular misalignment of the coils [80].

#### 3.2.3. AC Resistance

The AC resistance of a PSC is dominated by the dc resistance and is defined as [36,69,72]:

(13) $$R_{AC}=R_{skin}+R_{proximity}=R_{dc}\left(\begin{array}{c} \frac{t}{\delta \left(1-e^{-\frac{t}{\delta }}\right)}\cdot \frac{1}{1+\frac{t}{w}}+\frac{1}{10}{\left(\frac{\omega }{\frac{3.1\rho \left(s+w\right)}{\mu_{o}w^{2}t}}\right)}^{2} \end{array}\right)$$

where *ρ*, *t* and *s* are the resistivity, thickness and wire spacing, respectively. *R_dc_* and *δ* are the dc resistance and skin depth, respectively, so that:

(14) $$R_{dc}=\frac{\rho l}{wt}$$

(15) $$\delta =\sqrt{\frac{\rho }{\pi \mu f}}$$

where *l* is the complete wire length of the coil and *f* is the operating frequency. The commonly used formula for AC resistance of a WWC is given as [75,81,82]:

(16) $$R_{AC}=\frac{R_{dc}D_{o}^{2}}{4\delta \left(D_{o}-\delta \right)}$$

Another approach is to calculate iteratively the AC resistance of WWC using [65]:

(17) $$R_{AC}=R_{dc}\left(1+\frac{f^{2}}{f_{h}^{2}}\right)$$

where *f_h_* is the frequency at which the AC power dissipation is twice the dc power dissipation.

#### 3.2.4. Parasitic Capacitance

Different expressions are recorded in the literature for the PSC and WWC. The parasitic capacitance, *C_par_*, of a PSC for single dielectric layer is given as [36,72]:

(18) $$C_{par}=\frac{\left(\alpha \epsilon_{air}+\beta \epsilon_{substrate}\right)\epsilon_{o}tl_{g}}{s}$$

where *α* and *β* are empirical parameters determined form experiments, *l_g_* is the total length of the gap between two wires and *ε_air_* and *ε_substrate_* are the relative permittivity values of air and of the substrate. In [69], a detailed analysis of the parasitic capacitance is included by considering different dielectric layers around the WPT coil. The simplified form is written as:

(19) $$C_{par}=\epsilon_{r\_effective}\epsilon_{o}\frac{K\left(k^{\prime }\right)}{K\left(k\right)}\times l+C_{tov}$$

where *ϵ_r_effective_* and *C_tov_* are the effective relative dielectric constant and overlapping trace capacitance. *K* is the complete elliptic integral of the first kind and *k* (and $k^{\prime }$) are defined in [67]. From [65,75,82], the parasitic capacitance between two turns of a WWC, *C_t_*, is:

(20) $$C_{t}=\epsilon_{o}\epsilon_{r}\overset{\frac{\pi }{4}}{\underset{0}{\int }}\frac{\pi D_{x}r_{o}}{\varsigma +\epsilon_{r}r_{o}\left(1-\cos\theta \right)+0.5\epsilon_{r}s_{t}}d\theta$$

where *ς* is the thickness of the insulating wire, *r_o_* is the radius of the conducting section of the wire, *D_x_* is the average diameter of the insulating coating shell given by (*D_in_* + *D_c_*)/2 [83].

The parasitic capacitance between two layers, *C_l_*, can be written as:

(21) $$C_{l}=\epsilon_{o}\epsilon_{r}\overset{\frac{\pi }{4}}{\underset{0}{\int }}\frac{\pi D_{x}r_{o}}{\varsigma +\epsilon_{r}r_{o}\left(1-\cos\theta \right)+0.5\epsilon_{r}s_{l}}d\theta$$

A general expression for the total parasitic capacitance for multi-loop, multi-turn coil is:

(22) $$C_{self}=\frac{1}{N_{tot}^{2}}\left[\begin{array}{c} C_{t}\left(N_{t}-1\right)N_{l}+C_{l}\sum_{i=1}^{N_{t}}{\left(2i-1\right)}^{2}\left(N_{l}-1\right) \end{array}\right]$$

where *N_tot_* = *N_t_* × *N_l_*, with *N_t_* being the total number of turns and *N_l_* the total number of layers.

#### 3.2.5. PTE

The PTE can be estimated by utilizing different theories in the literature. Using lumped element circuit analysis, the simplified expression of the PTE, *η_ij_*, between the coils *C_i_* and *C_j_* can be written as [84,85,86]:

(23) $$\eta_{ij}=\frac{k_{ij}^{2}Q_{i}Q_{j}}{1+k_{ij}^{2}Q_{i}Q_{j}}$$

where *k_ij_* ($=M_{ij}/\sqrt{L_{i}L_{j}}$) is the coupling coefficient between coils, *C_i_* and *C_j_*. *Q_i_* and *Q_j_* are the quality factor of *C_i_* and *C_j_*, respectively. Without the effect of load resistance *Q_i_* and *Q_j_* are known as unloaded quality factor, *Q_unloaded_* and defined as:

(24) $$Q_{unloaded}=\frac{\omega L-\omega \left(R_{AC}^{2}+\omega^{2}L^{2}\right)C_{par}}{R_{AC}}$$

where *ω* is the radial frequency of operation and *L* is the total inductance of the coil. The expression of PTE using coupled mode theory (CMT) [75,79,82] is:

(25) $$\eta_{ij}=\frac{1}{1+\frac{Q_{L}}{Q_{j}}\left[1+\frac{1}{FOM^{2}}{\left(1+\frac{Q_{j}}{Q_{L}}\right)}^{2}\right]}$$

where *Q_L_* (=2*πfL_RX_*/*R_L_*) [15,87,88] is the loaded Q-factor calculated by using the inductance of the RX coil, *L_RX_*, and the load resistance, *R_L_,* and the dimensionless figure-of-merit, *FOM*, defined as [87]:

(26) $$FOM=\sqrt{k_{i,j}^{2}Q_{i}Q_{j}}$$

The PTE can also be derived using reflected load theory (RLT) [75,76,82]:

(27) $$\eta_{ij}=\frac{k_{ij}^{2}Q_{i}Q_{jL}}{1+k_{ij}^{2}Q_{i}Q_{jL}}\frac{Q_{jL}}{Q_{L}}$$

where *Q_jL_* is the loaded Q-factor of *Q_j_*, where *j* represents the number of the coil. The CMT and RLT equations of PTE can be shown to be mathematically identical in steady state conditions [60].

#### 3.2.6. Optimization Flow

PTE is the primary evaluation metric for NRIC WPT link. Its performance is commonly limited by the skin effect and proximity effect resistances [36,69,72], large separation distance between TX and RX, translational and angular misalignment of the coils and tissue losses. Furthermore, the selection of the operating frequency to maximize the coils quality factor, proper impedance matching of the two-port network and optimal load resistance value can contribute to maximum PTE for this WPT scheme.

**Figure 5:**
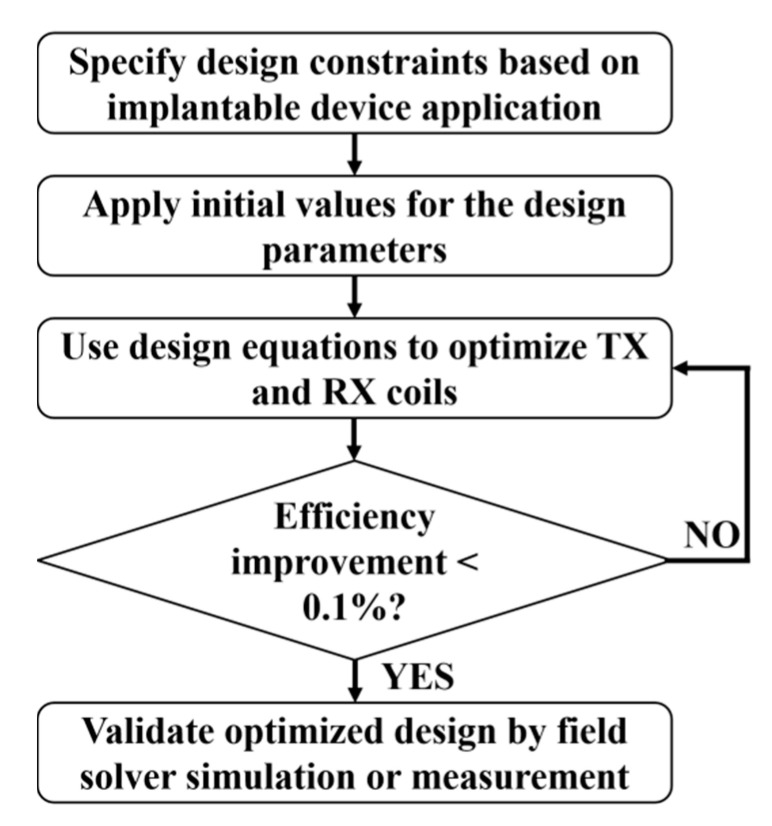
Optimization flow graph of NRIC WPT system.

As shown in Figure 5, an iterative optimization flow diagram has been adopted in most of the research literature to achieve maximum PTE [36,66,70,75,76]. A nested multidimensional optimization algorithm has recently been proposed in [79]. This algorithm can process the various design parameters in a multidimensional design space simultaneously and reach the global maximum efficiently. In general, the frequency of operation of the NRIC is limited to hundreds of kHz to a few tens of MHz.

### 3.3. System Design

**Figure 6:**
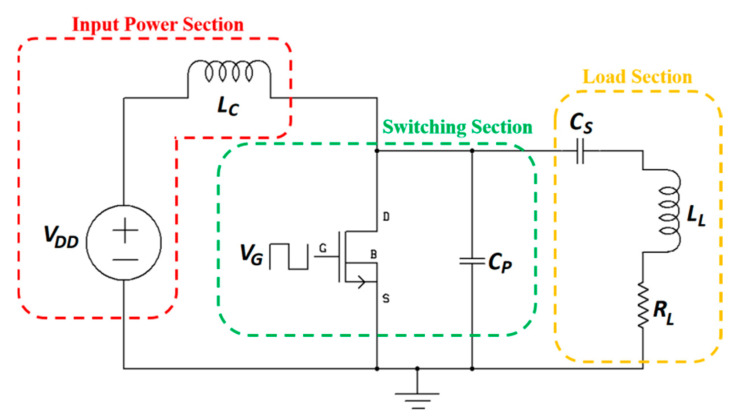
Single-ended class-E power amplifier (PA) for NRIC WPT.

Figure 6 shows the circuit topology of a single-ended class-E PA also known as a DC-fed energy injection converter.

In this topology, the resonant circuit is used as an energy storage element [89]. The input power section is composed of a DC voltage source, *V_DD_*, and a choke inductor, *L_C_*. The switching section includes a MOSFET switch driven by the input voltage *V_G_* generated from the MOSFET driver integrated circuit and operated at the WPT frequency, and a parallel capacitor, *C_P_*. Finally, the load section contains in series the resonating capacitor, *C_S_*, and the load (*L_L_*, *R_L_*), where *L_L_* is the TX coil of the NRIC link. Besides the nominal-based class-E PA [90,91,92], a higher efficiency sub-nominal class-E PA is presented in its general context [93] and for MIDs [94]. The amplifier, operated at 1 MHz frequency, is composed of the MOSFET IRF640N switch with an IXDN414 driver. The highest achieved efficiency was 99.3% for a 0.097 duty ratio, and 91% for the more common 0.5 duty ratio [94].

For a given *L_L_*, the optimized *C_S_* can be calculated as:

(28) $$C_{S}=\frac{1}{2\pi f\left(2\pi fL_{L}-X_{L}\right)}$$

where *X_L_* is the reactance of the load loop [94]. Finally, *C_P_* can be optimized and given as:

(29) $$C_{P}=\frac{X}{8\pi^{3}fX_{L}\left(1-\delta \right)}$$

where *X* is defined in [94]. An extensive review of different TX circuit topologies for NRIC is presented in [95].

**Figure 7:**
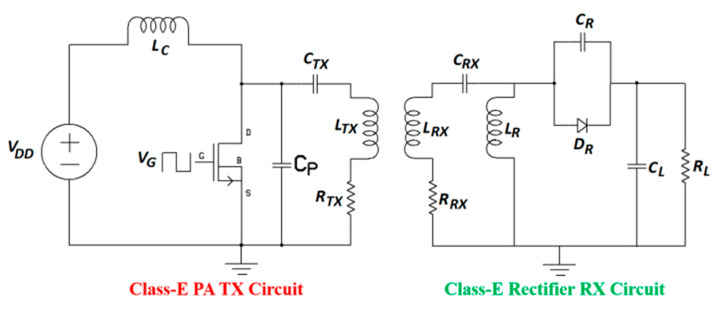
Schematic of class-E PA and rectifier system for NRIC.

Figure 7 shows a nominal [93] class-E PA and class-E current driven rectifier [96]. The system efficiency is recorded as 84% after rectification for an operating frequency of 6.78 MHz. Although proposed for a generic NRIC WPT system, this topology can be adopted for MID applications. The rectifier consists of a Schottky diode, *D_R_* (STPSC406), a parallel capacitor, *C_R_*, a filter capacitor, *C_L_*, a filter inductor, *L_R_*, and a dc load, *R_L_*. *L_TX_* and *L_RX_* are the inductances; *R_TX_* and *R_RX_* are the AC resistances; and *C_TX_* and *C_RX_* are the series resonant capacitances of the TX and RX coils, respectively. A detailed analysis and optimization of the parameters is provided in [96].

A similar rectifier topology with Schottky diodes is presented in [97,98] for three dimensional (3D) NRIC WPT for MIDs. A series rectifier architecture is adopted in this work for multiple rectifiers for 3D coils. Parallel rectifier topologies have also been published for 3D NRIC WPT [99,100,101]. The output power of series rectifier topology is higher than that of the parallel topology as all the rectifiers contribute to total power. In the parallel rectifier architecture, only the rectifier that produces the maximum voltage contributes to the total output power. However, the series rectifier topology increases the power loss from Schottky diodes compared to a parallel architecture. Efficiency analyses of the Schottky diode-based rectifiers are rare in the literature. However, the efficiency of this type of rectifier varies between 30% to 80% and significantly lower at higher operating frequencies [102] due to an increase in leakage current. Therefore, the lower efficiency of the rectifier can reduce the overall system efficiency of the WPT. To overcome this challenge several application specific integrated circuit (ASIC)-based high efficiency rectifiers have been proposed in offering 93.6% [103], 87% [104] and 86% [105] efficiencies for MIDs.

### 3.4. Applications

#### 3.4.1. Brain Implant

**Figure 8:**
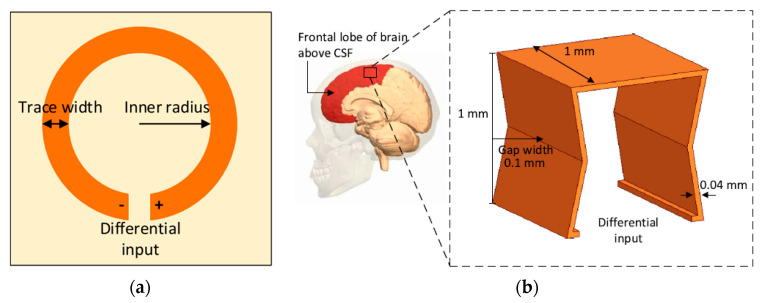
Geometry of the proposed NRIC WPT link [106]. (**a**) TX coil printed on FR4 board. (**b**) RX three-dimensional (3D) antenna embedded in the cerebral spinal fluid (CSF) above the frontal lope of the brain. Copyright © 2018, IEEE.

A NRIC WPT link for brain implant has recently been proposed in [106]. Figure 8a shows the TX coil printed on the 3.2 mm thick FR4 substrate of *ϵ_r_* = 4.3. The proposed 3D RX coil has an inner gap width of 0.1 mm and a total volume of 0.9 mm^3^, as shown in Figure 8b. The inner radius and the trace width are 6 and 3 mm, respectively, for a single layer TX. The operational frequency is 402 MHz.

**Figure 9:**
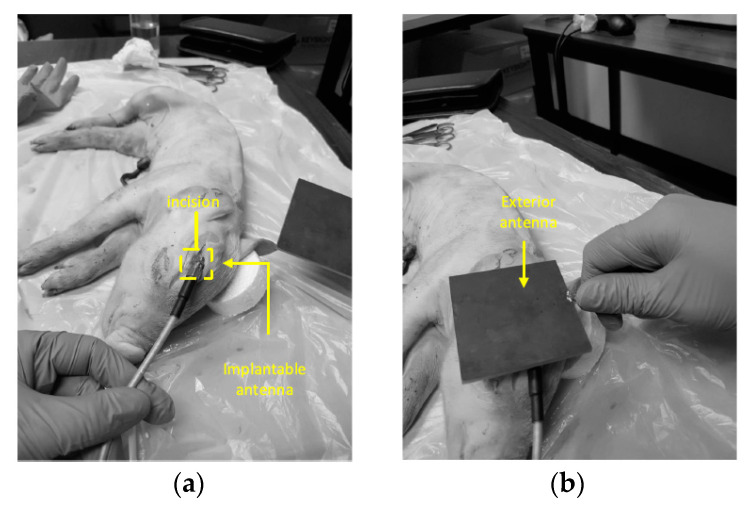
Measurement of the proposed NRIC WPT link for a piglet [106]. (**a**) An incision was created in the skull to embed RX coil over the brain. (**b**) Position of the external TX coil. Copyright © 2018, IEEE.

This proposed NRIC WPT link was tested inside the head of a pig and piglet to confirm the performance of the designed link in biological environment, as shown in Figure 9a,b. The maximum efficiency achieved by the system was measured at 0.7%, 0.02% and 0.08% in air, pig and piglet, respectively. The maximum SAR was 1.97 W/kg for 10 g of tissue and 82 mW input power [106].

**Figure 10:**
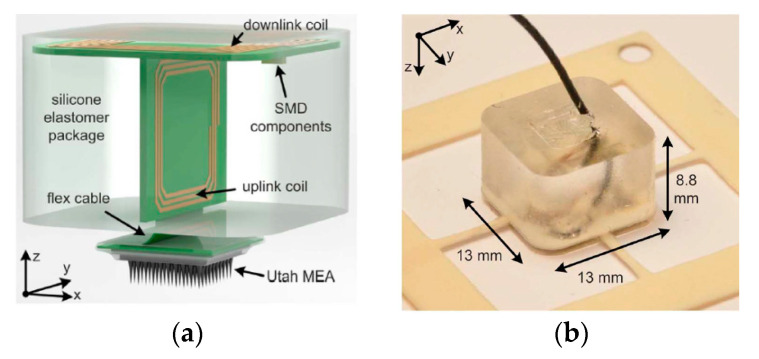
Proposed two-body packaging for a wireless cortical implant [107]. (**a**) Components of the package. (**b**) Fabricated package. Copyright © 2011, IEEE.

A new packaging strategy for the WPT system was proposed using biocompatible materials such as polydimethylsiloxane (PDMS) and Parylene-C [107]. Figure 10a shows the transceiver part of the silicone elastomer package (Sylgard 184 PDMS, Dow corning) coated with a 2 μm thick layer of Parylene-C to improve its biocompatibility [108,109,110]. Figure 10b shows the fabricated transceiver package of dimensions 13 × 13 × 8.8 mm^3^ including the implant inductive link. The maximum measured link efficiency is 54.98% at 8 MHz and 10 mm separation distance in air [69]. Link efficiency was not measured in the biological tissue.

**Figure 11:**
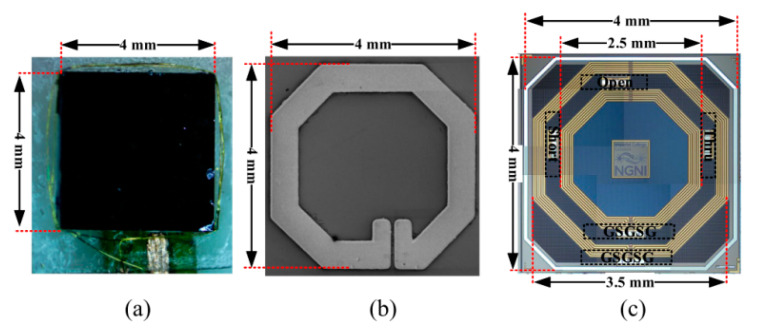
Chip-scale RX coils [40]. (**a**) around-CMOS. (**b**) above-CMOS. (**c**) in-CMOS. Copyright © 2018, IEEE.

Chip-scale coils have been proposed for implantable neural microsystems [40]. The RX coils were wire-wound around the Complementary Metal Oxide Semiconductor (CMOS) die of outer diameter of 4 mm, as shown in Figure 11a. Other CMOS chips were post-processed to accommodate the coils on top of the CMOS die of 2 mm diameter, as shown in Figure 11b. A third version contains the coils fully integrated in the CMOS die of 4 mm diameter as shown in Figure 11c. The track width of the around, above and in-CMOS coils are 25, 250 and 175 μm, respectively. Single turn TX coils were designed individually for the three types of RX coils in air and tissue. The maximum PTE achieved was 3.05% for the around-CMOS RX coil and a 17.2 mm diameter TX coil at a frequency of 318.8 MHz. Measurements were carried out ex vivo on 11 mm lamb ribs. A maximum SAR of 0.155 W/kg for 10 mW of transmitting power was recorded for the around-CMOS RX coil.

#### 3.4.2. Neurostimulator Implants

**Figure 12:**
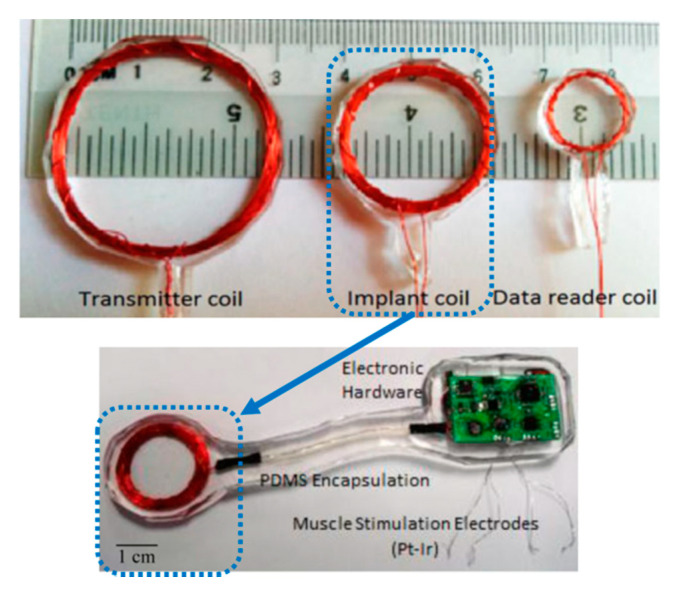
Different coils and stimulation implant used in the rat experiment [111]. Copyright © 2015, IEEE.

This implant is designed to generate patterned stimulation to re-establish the motor and sensory function in the limbs. The implant can differentiate the nerve signals by recording and transferring the signals wirelessly to the stimulator implants. It can therefore assist to convey the nerve signals to the denervated muscles and restore functionality immediately. Figure 12 shows the stimulator implanted through a small incision made in the stomach region of a rat [111]. The wire wound RX coils are encapsulated with PDMS. The TX and RX coils diameters are reported as 30 mm and 20 mm, respectively. A PTE of 65.8% as measured in vivo for a 1 MHz operation frequency and 5 mm separation distance between the TX and RX coils. The SAR for 180 mW TX power was calculated at 0.1 W/kg.

**Figure 13:**
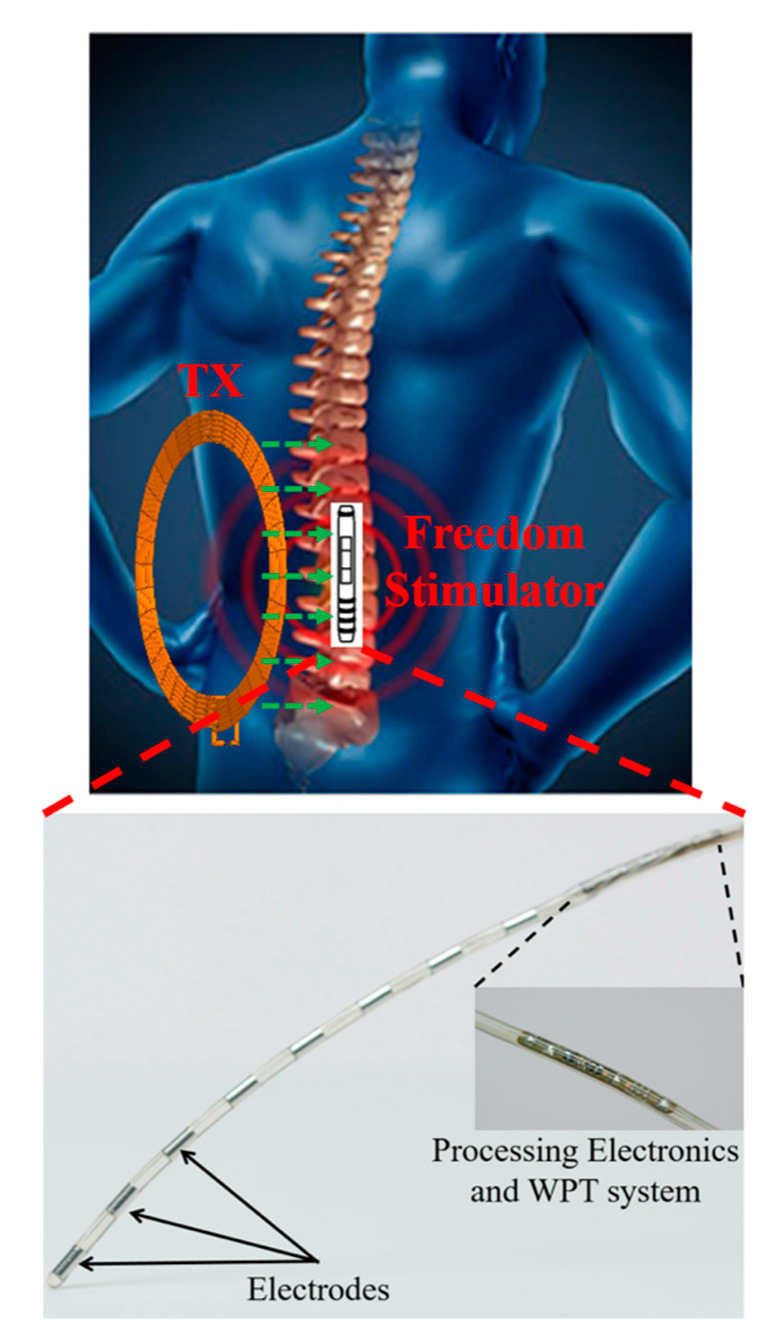
Spinal cord stimulator for StimWave.

Figure 13 shows the first FDA approved spinal cord stimulation implant (Freedom System^TM^) by StimWave. It is a set of thin wires and a micro-receiver covered in a protective casing. The neurostimulator has small metal electrodes near the tip that can create electrical field energy when power is applied. The pain signals coming from certain nerves of the spinal cords are blocked. This neurostimulator receives WPT from an external module.

#### 3.4.3. OcularImplant

**Figure 14:**
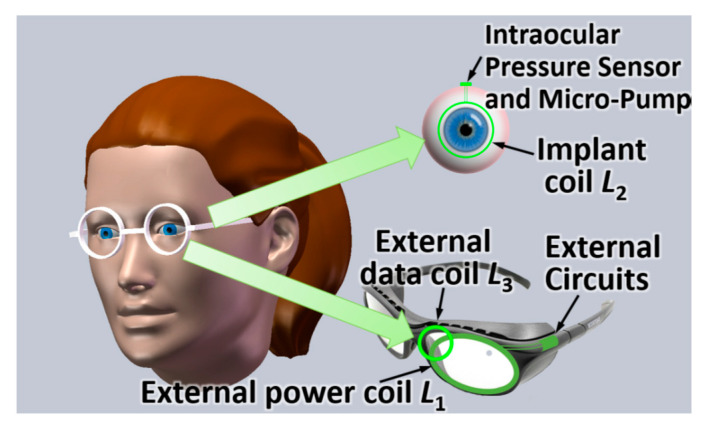
Proposed intraocular sensor system for glaucoma treatment [112]. Reproduced with permission from the corresponding author and MDPI Sensors.

In [112], an inductive link is proposed for glaucoma treatment with an additional coil for the uplink reception of data. Figure 14 shows an artist impression of the intraocular biomedical sensor device. The device is to be located on the patient’s eyeball to monitor and regulate the intraocular pressure for glaucoma treatment. Three coils are used for wireless power and data transfer. The external power coil (*L*_1_), external data coil (*L*_3_) and implant coil (*L*_2_) are used to feed wireless power, receive uplink data, and both harvest wireless power and transmit uplink data, respectively. The outer diameter of *L*_1_, *L*_2_ and *L*_3_ are 40 mm, 20 mm and 16 mm, respectively. The WPT link frequency is 2 MHz.

**Figure 15:**
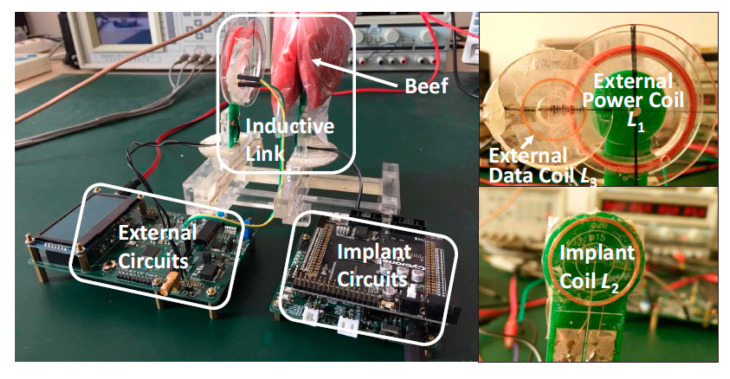
Prototype and measurement setup of the proposed system [112]. Reproduced with permission from corresponding author and MDPI Sensors.

Figure 15 shows the measurement setup of the proposed system [112]. The high measured PTE of 5% for a maximum coil separation distance of 40 mm is likely to be due to the high quality factor of the coil, the large size of the TX and the minimum presence of tissue between TX and RX. The authors claimed the same PTE for the air and beef muscle medium due to the usage of lower frequency of power transfer link. The maximum local SAR is 0.66 W/kg.

**Figure 16:**
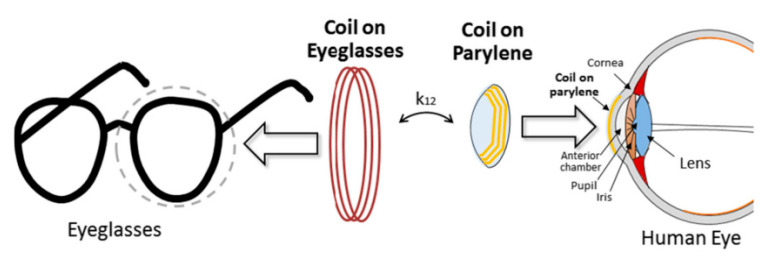
Proposed system [113]. External coil is embedded to eyeglasses. A thin and small implantable coil is embedded on a wearable Parylene platform. Reproduced with permission from Springer Nature (Journal: Biomedical Microdevices), Copyright © 2015.

**Figure 17:**
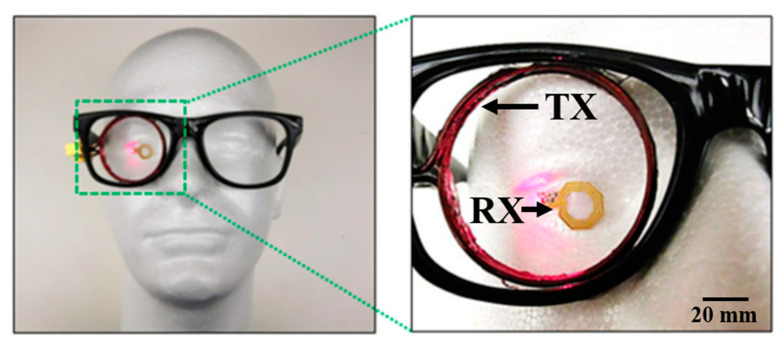
Concept demonstration of eyeglass-powered contact lens [113]. Reproduced with permission from Springer Nature (Journal: Biomedical Microdevices), Copyright © 2015.

Another ocular diagnostics device embedded into a contact lens [113] and fabricated on a Parylene substrate is shown in Figure 16 and Figure 17. The external coil is embedded into an eyeglass. The operation frequency of the system is 13.56 MHz. The outer diameter of the TX and RX coil is 45 mm and 10 mm, respectively. A PTE of 17.5% is achieved on a pig eye at a 20-mm of separation distance. The peak local SAR is 0.021 W/kg for 2 W of input power.

**Figure 18:**
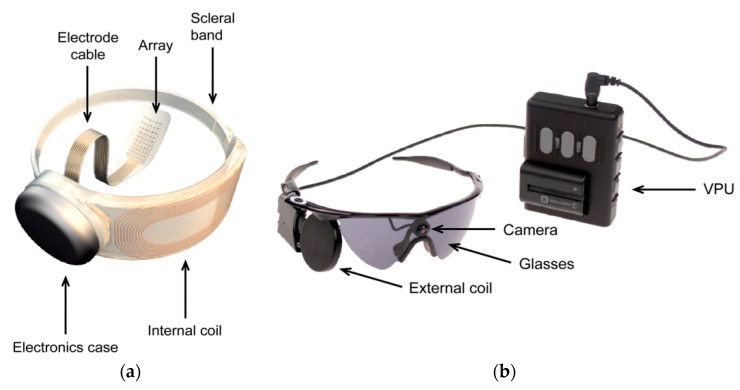
ARGUS II retinal prosthesis system [114]. (**a**) Wearable external parts. (**b**) Illustration of the implanted parts. Copyright © 2018 Second Sight Medical Products, Inc, USA.

A commercial retinal prosthesis system [114,115], ARGUS II, developed by Second Sight Medical Products, is presented in Figure 18a,b.

The epiretinal implant system, shown in Figure 18a, consists of an RX coil, processing electronics, 6 × 10 platinum electrode array to electrically stimulate the retinal neurons and a scleral band for the positioning of the implant around the eye. The external part of the system is shown in Figure 18b and consists of a miniature video camera, a TX coil attached and a video processing unit (VPU). The retinal implant is powered using the same NRIC link as the data at the frequency of 3.156 MHz.

#### 3.4.4. Cochlear Implant

**Figure 19:**
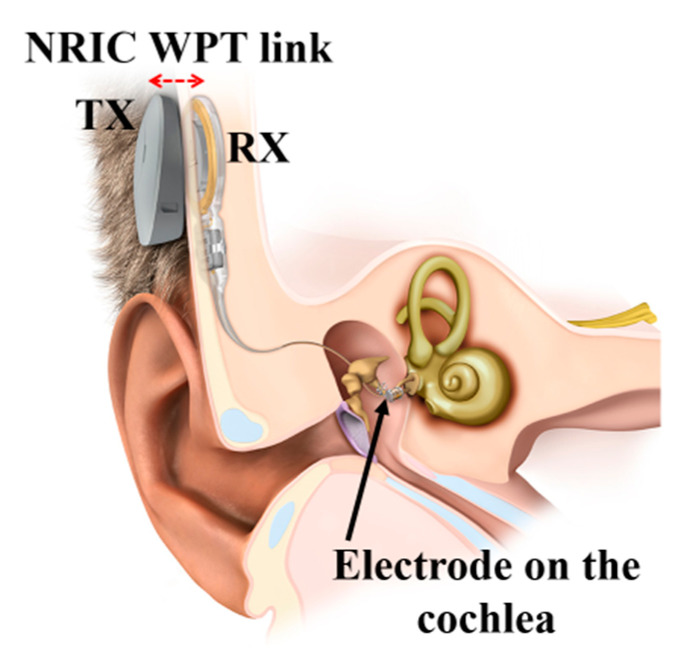
MED-EL cochlear implant NRIC WPT link. Adopted with permission from MED-EL.

Figure 19 shows a commercially available, FDA-approved, NRIC WPT link-based cochlear implant device manufactured by MED-EL [116]. A permanent magnet was used in the center of the coils to align the magnetic fluxes emanating from and to the TX and RX coils, respectively.

Unfortunately, no further information regarding the operating frequency, coil parameters, link transfer efficiency and overall PTE is available.

#### 3.4.5. Capsule Endoscopy

Capsule endoscopy (CE) promises early stage minimally invasive detection and rapid diagnosis of GI diseases [117] to limit the adverse effects of gastrointestinal (GI) disorders [118,119]. Many researchers today work towards WPT solutions to supply sufficient power to support multi-modality functions of future CE. In [99], an NRIC power receiver for CE comprises three geometrically orthogonal coils to ensure a strong coupling with the TX for any orientation of the RX. The RX is a WWC made of 100 µm diameter copper wire, and has a cylindrical shape with outer dimensions of 10 × 13 mm^2^. The operation frequency was chosen as 1 MHz. The inner diameter of the TX is 41 cm and is made of rectangular Litz cable [120]. The worst-case PTE is reported as 1% in air for an SAR of 0.32 W/kg at approximately 20 cm separation distance with the TX.

**Figure 20:**
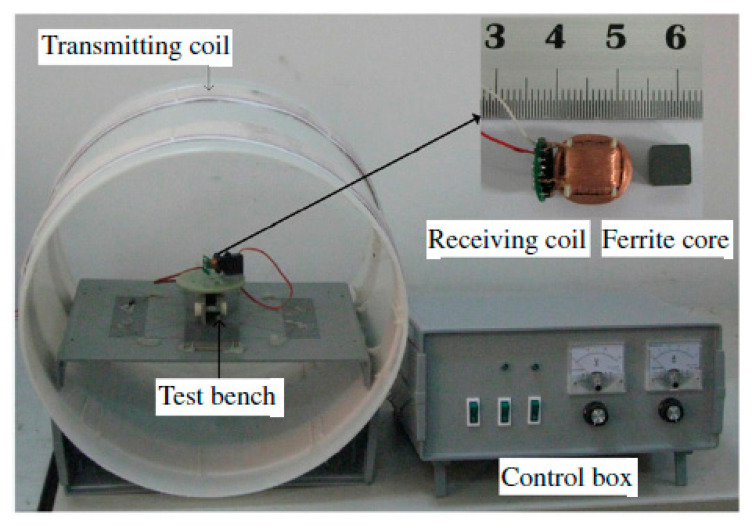
Experimental setup of optimized 3D receiver [121]. Copyright © Institute of Physics and Engineering in Medicine. Reproduced with permission of IOP Publishing.

**Figure 21:**
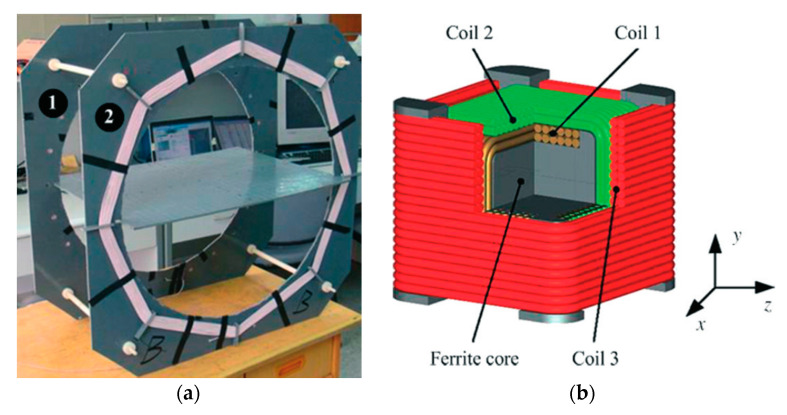
3D WPT system for capsule endoscopy (CE) [122]. (**a**) Helmholtz TX coil. (**b**) Structure of the 3D receiver. Reproduced with permission from John Wiley and Sons, Copyright © 2010.

Figure 20 presents the experimental setup of a system operated at 218 kHz [121]. The novel TX coil structure is composed of a pair of double-layer solenoids of 40 cm diameter with 180 strands of AWG38 enameled copper wire with 25 turns for each layer, and wound on an acrylonitrile butadine styrene (ABS) hollow cylinder. The RX coil is fabricated with Litz wire AWG44 surrounding a high permeability, 6.6 × 6.6 × 6.6 mm^3^ MnZn ferrite cylinder. The RX coil has a diameter of 11.5 mm for a length of 11.5 mm. The received power at the load is 540 mW for PTE of 5.5% in air. The SAR reported for 200 kHz and 1.8 A drive current is 8 W/kg for 10g of tissue. In [122], an RX structure similar to the one in [121] is presented at operating frequency of 400 kHz. Figure 21a shows two Helmholtz TX coils with an outer diameter of 69 cm that are oriented parallel to one another. The outer dimensions of the receiver coil are 10 mm length for a 12 mm diameter. In this 3D receiver, the coils 1, 2 and 3 are geometrically orthogonal to each other and wound on a common ferrite core, as shown in Figure 21b. The received power at the load is 310 mW for a link PTE of 1.2% in air and an SAR of 0.329 W/kg.

**Figure 22:**
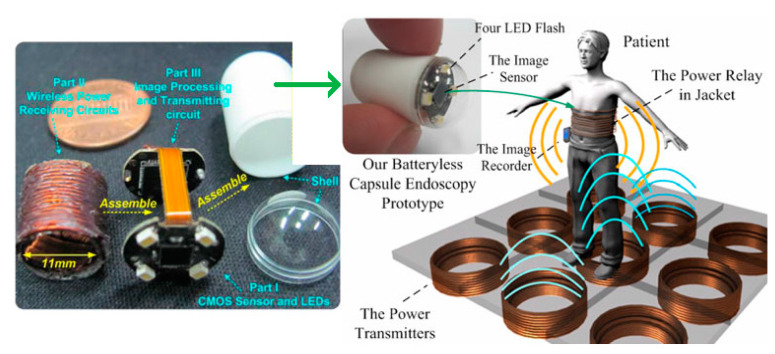
Two-hop NRIC WPT CE system [103]. Copyright © 2012, IEEE.

In [103], the PTE of the CE is improved by using a two-hop-based NRIC TX system and switch mode rectifier circuit. Figure 22 shows the proposed system where the PTE was measured at 3.04% using an in vitro human phantom model filled with saline water. The receiver size is 11 mm in diameter for a length of 27 mm. The maximum SAR is 0.1 W/kg for an operating frequency of 13.56 MHz and 8 W of input power.

### 3.5. Design Challenges and Future Trends

NRIC is the most established WPT method for MIDs. Issues associated with the implementation of NRIC WPT link include:

(1) Optimization only for a particular load to achieve maximum power. Hence, the operation of such a link to extract efficient power in variable load conditions still remains to be resolved.
(2) Motion of MIDs can cause misalignment in the RX coil and reduce the efficiency of the WPT link. Therefore, recent research has focused on the 3D orthogonal WPT receiver architecture to mitigate the misalignment effect [123].
(3) Robustness of performance of flexible implantable NRIC coils is another important design challenge. Severe mismatch of the resonance capacitance can occur due to the flexion characteristics of the implant coils [124]. Therefore, self-tuning circuitry is required to keep the transfer efficiency stable. Some attempts have been reported to address the self-tuning of the NRIC WPT resonators [125,126].
(4) The large power requirement of some multi-functional implantable devices might prohibit the use of a WPT system that could generate an SAR above 2 W/kg, the recommended limit regarding safety of human tissues [117,127]. It is unfortunate that most researchers fail to measure the SAR of their designed coils.
(5) Biocompatibility of the material used for the implanted coil is not published widely in the literature. This information is of critical importance to facilitate medical acceptability of the implant.

The demand for miniaturized MIDs has led to an increase in research on the development of chip-scale-based [40,128] NRIC where the WPT coils are embedded with the integrated circuits (ICs). Furthermore, alternative approaches, such as 3D orthogonal structures [99,129,130,131] and ferrite materials [132] have attracted the attention of researchers as a means of improving the PTE. Recent research regarding the use of antiparallel resonant loops [133,134] demonstrate a higher PTE at a larger separation distance. This technique does not require complex impedance matching network associated with traditional WPT coils. Therefore, this technique appears to be a potential future candidate for improving the efficiency of the NRIC link.

### 3.6. Verdict

NRIC is one of the most studied WPT techniques for applications, including IMD. Therefore, this technique offers power supply solutions to a wide range of IMDs. A systematic design and optimization technique is present in the literature for NRIC. Furthermore, the lower MHz operation frequency range offers comparatively higher tissue safety than with other WPT techniques. The simple coil architecture of NRIC is easy to manufacture. It also offers a relatively higher PTE for the IMDs. Misalignment of the RX coil is a significant issue for NRIC as it drastically affects the PTE. However, recent research on 3D WPT coils demonstrated it is a promising solution for the misalignment issue of NRIC [80,135]. Therefore, multiple commercial IMD manufacturers are adopting NRIC as an alternative solution to batteries.

## 4. Non-Radiative Magnetic Resonance Coupling

### 4.1. Link Design

**Figure 23:**
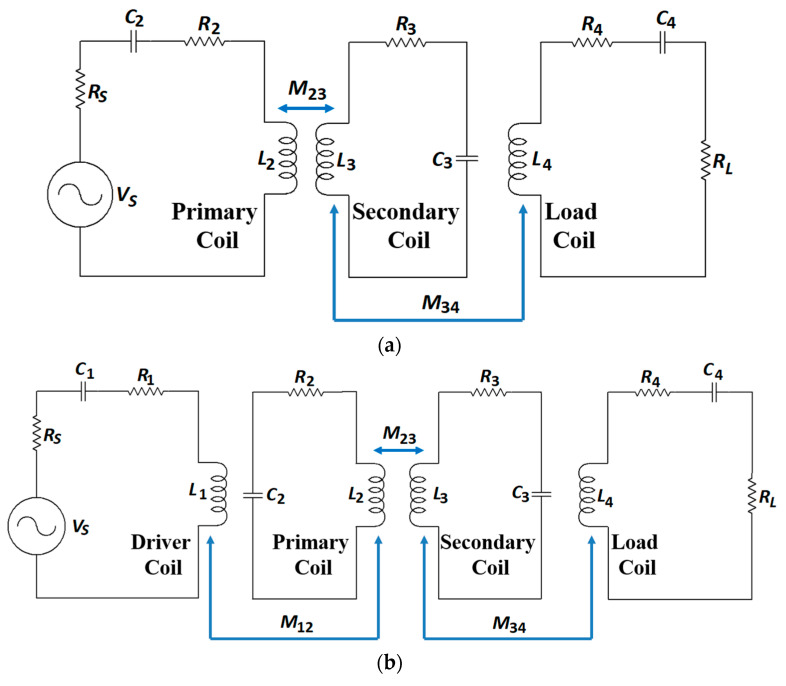
Non-radiative magnetic resonance coupling (NRMRC) equivalent circuit. (**a**) Three-coil technique. (**b**) Four-coil technique.

Non-radiative magnetic resonance coupling (NRMRC), also known as strongly coupled magnetic resonance (SCMR) [63], was proposed by researchers from MIT in 2007 [15]. Compared with the NRIC method, the NRMRC technique uses three [136] or four coil-based [137] architectures, as shown in Figure 23a,b, respectively, depending on the practical requirements, such as separation distance, PTE and load power rating [63].

The three-coil-based WPT system has one TX coil, which is the primary coil, and two RX coils, defined as the secondary and load coils [87]. Depending on a specific set of electrical parameters, the load can be made electromagnetically transparent to the driving circuitry ensuring maximum power transfer [138]. In [75], a comprehensive analysis of the method concluded that three coils could increase power transfer efficiency compared to four coils and simultaneously keep the transfer efficiency constant over changes in the transferred power [63]. The four-coil-based configuration consists of two TX coils, defined as the driver and primary coils, and the RX secondary and load coils. The driver and load coils can be used for impedance matching [139].

Both configurations have superior performance characteristics than the NRIC in several aspects: (a) better impedance matching capability to optimize the system power transfer, (b) higher Q-factor enabled by the primary and secondary coils, which can compensate for the sharp decline of PTE caused by the reduced coupling coefficient due to the increasing separation distance and (c) higher bandwidth of operation [137].

### 4.2. Optimization

The optimization methodology of NRMRC follows a similar approach to NRIC. In [67,140,141] and [137,142], *L* is calculated using Equations (6) and (8), and *M_ij_* is determined using (9) and (11). The calculation of the AC resistance and parasitic capacitance of WWCs uses Equations (17) and (22), respectively [115,116,122]. For PSCs, the same parameters are calculated using Equations (13), (18) and (19) [67,140,141]. The PTE of the three-coil-based system using lumped element analysis is derived as [136,143]:

(30) $$\eta_{3-coil}=\frac{\left(k_{23}^{2}Q_{2}Q_{3}\right)\left(k_{34}^{2}Q_{3}Q_{4L}\right)+\left(k_{24}^{2}Q_{2}Q_{4L}\right)}{\left(1+k_{34}^{2}Q_{3}Q_{4L}\right)\left(\begin{array}{c} 1+k_{23}^{2}Q_{2}Q_{3}\\ +k_{34}^{2}Q_{3}Q_{4L}+k_{24}^{2}Q_{2}Q_{4L} \end{array}\right)}$$

where *k_ij_* and *Q_i_* are the coupling coefficients between coils *C_i_* and *C_j_*, and the Q-factor of coil *C_i_*, respectively. In [75,82], the PTE of three-coil-based NRMRC WPT system is analysed using RLT and simplified as:

(31) $$\eta_{3-coil}=\frac{\left(k_{23}^{2}Q_{2}Q_{3}\right)\left(k_{34}^{2}Q_{3}Q_{4L}\right)}{\left(1+k_{34}^{2}Q_{3}Q_{4L}\right)\left(\begin{array}{c} 1+k_{23}^{2}Q_{2}Q_{3}\\ +k_{34}^{2}Q_{3}Q_{4L} \end{array}\right)}\frac{Q_{4L}}{Q_{L}}$$

where *Q_L_* is the load quality factor (=*R_L_*/2*πfL*) and *Q_iL_* = *Q_i_Q_L_*/(*Q_i_* + *Q_L_*) [82]. The PTE of four-coil-based NRMRC WPT system using LCT is given as [137,142,144]:

(32) $$\eta_{4-coil}=\frac{\left(k_{12}^{2}Q_{1}Q_{2}\right)\left(k_{23}^{2}Q_{2}Q_{3}\right)\left(k_{34}^{2}Q_{3}Q_{4}\right)}{\left[\begin{array}{c} \left(1+k_{12}^{2}Q_{1}Q_{2}\right)\\ \left(1+k_{34}^{2}Q_{3}Q_{4}\right)+k_{23}^{2}Q_{2}Q_{3} \end{array}\right]\left(\begin{array}{c} 1+k_{23}^{2}Q_{2}Q_{3}\\ +k_{34}^{2}Q_{3}Q_{4} \end{array}\right)}$$

The PTE of four-coil-based NRMRC WPT system using RLT can be written as [67,75,82,141]:

(33) $$\eta_{4-coil}=\frac{\left(k_{12}^{2}Q_{1}Q_{2}\right)\left(k_{23}^{2}Q_{2}Q_{3}\right)\left(k_{34}^{2}Q_{3}Q_{4L}\right)}{\left[\begin{array}{c} \left(1+k_{12}^{2}Q_{1}Q_{2}\right)\\ \left(1+k_{34}^{2}Q_{3}Q_{4}\right)+k_{23}^{2}Q_{2}Q_{3} \end{array}\right]\left(\begin{array}{c} 1+k_{23}^{2}Q_{2}Q_{3}\\ +k_{34}^{2}Q_{3}Q_{4L} \end{array}\right)}\frac{Q_{4L}}{Q_{L}}$$

The optimization of NRMRC follows a similar methodology as the one presented in Figure 5 [67,75,82,137,140,141].

### 4.3. System Design

Similar TX and RX circuits as those used for the NRIC method can be used for NRMRC WPT systems, as all the coils are tuned to the same resonant frequency. However, during the design of the TX circuit, the reflection from all the coils needs to be considered.

### 4.4. Applications

#### 4.4.1. Brain Implant

**Figure 24:**
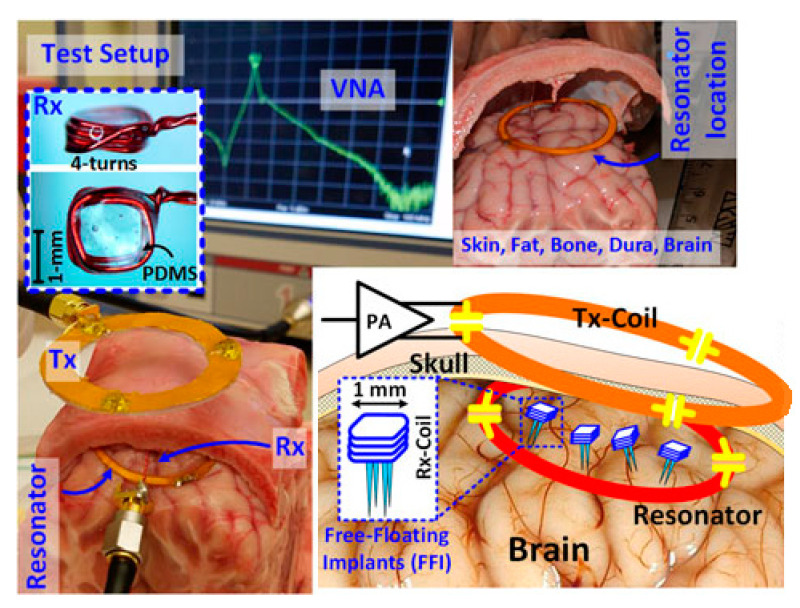
The three-coil link test setup, including a sheep brain and skull [145]. Copyright © 2017, IEEE.

Free-floating implants (FFIs) are presented in [145] for brain implant applications. For very small-sized RX coils, a high Q-factor intermediate resonator can significantly improve the PTE, which is explained analytically in [145]. Therefore, a three-coil based architecture operated at 60 MHz was adopted in this article. The outer diameters of the TX coil, intermediate resonator and RX coil are 45 mm, 32 mm and 1.2 mm, respectively. Figure 24 shows the PTE measurement setup where a 6 × 7 × 8 cm^3^ hole through the skull of a recently deceased lamb is used as an in vitro model. The sheep brain was refrigerated for around 24 h and kept outside for 1–2 h before the experiment in order to reach the room temperature. The measured average PTE was 3% for a 16 mm separation distance. The average SAR was less than 1.6 W/kg for 1 g of human head tissue mass.

#### 4.4.2. Ocular Implant

**Figure 25:**
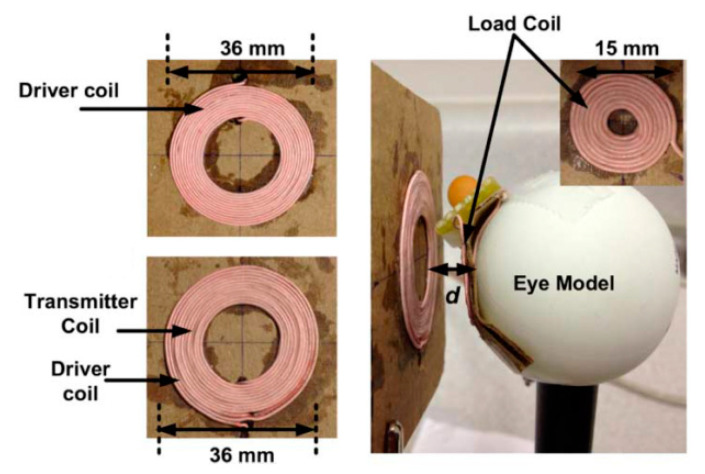
Eye model for three-coil WPT performance evaluation [146]. Copyright © 2012, IEEE.

In [146], a three-coil WPT system was proposed for ocular implants to improve the system tolerance to variations of the coupling factor. Figure 25 shows the hand-wound WPT coils and a 36 mm diameter plastic sphere that mimics the eye. The TX and RX coils of 36 mm and 15 mm outer diameter, respectively, are composed of AWG44 Litz wire. A maximum achievable PTE of 62.5% was achieved for a resonant frequency of 3.37 MHz at 10 mm of separation distance in air.

**Figure 26:**
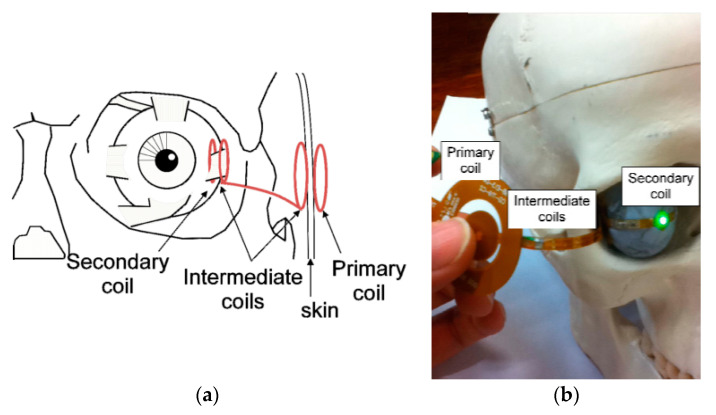
Multi-coil WPT retinal prostheses [147]. (**a**) Possible coil locations. (**b**) Measurement setup of two-pair coils system. Copyright © 2011, IEEE.

A two pair resonating coil (four-coil-based configuration) for retinal prosthesis was proposed in [147] for an operating frequency of 6.78 MHz. Figure 26a shows the coil locations inside the eye. Two intermediate coils are positioned with one end on the sclera over the secondary coil and the other end under the skin beneath the primary coil. The TX and RX coils are fabricated using flexible substrate technology. Figure 26b shows the placement of the coils on a human head model. The separation distance between TX and RX coil is 5 mm. The maximum achievable efficiency of the proposed system [147] is 8.8%, and the average load power is 50.2 mW.

#### 4.4.3. Capsule Endoscopy

**Figure 27:**
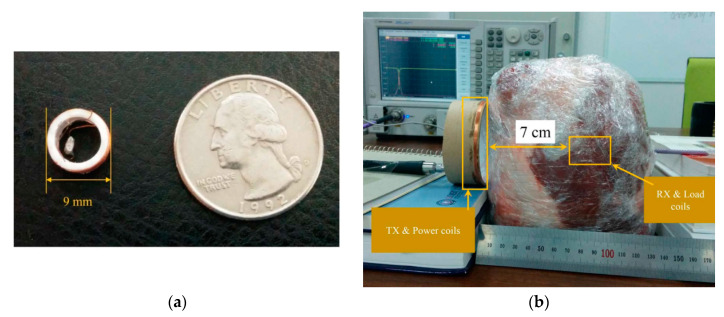
NRMRC WPT system for CE [148]. (**a**) Designed receiver coil with 9 mm diameter. (**b**) Experiment setup with a pork chop. Copyright © 2015, IEEE.

Figure 27a shows a four-coil WPT system for CE [148].The RX coil is 9 mm in diameter, and the cross-sectional diameter of the copper wire is 0.32 mm. The TX coil is 22 cm in diameter and has six turns. The resonance frequency of the TX and RX coils was selected as 16.47 MHz, which leads to an SAR of 1.74 W/kg. The transmitted power is 158 W, while the received power is measured at 26 mW in one experiment. The system described in [148] is tested in a 7 cm pig tissue medium, as shown in Figure 27b achieving a maximum PTE of 0.7%.

**Figure 28:**
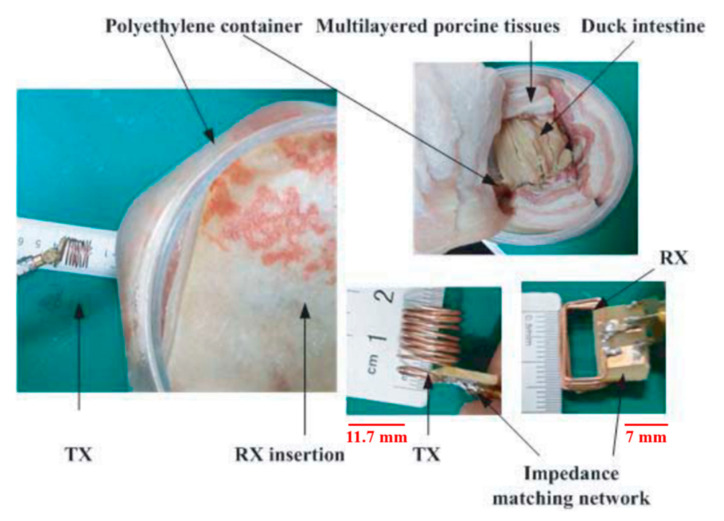
NRMRC WPT sub-GHz experimental setup [149]. Reproduced with permission of Electromagnetics Academy.

In [149], a 433.9 MHz resonance frequency WPT system is proposed, where the dimensions of the cylindrical TX is 11.71 mm in length for a diameter of 12.5 mm. The RX coils size is 15 × 7 × 6 mm^3^, as shown in Figure 28. The PTE is claimed to be 1.21% for a 5 cm separation distance from the duck intestine. The TX is placed 2 cm away from the skin, and the RX is positioned 3 cm inside the duck intestine. Furthermore, the SAR is 2.54 W/kg for 1 W of input power.

### 4.5. Design Challenges and Future Trends

Compared with NRIC, NRMRC is still under development but has been the focus of much attention from researchers over the last decade. The important design issues to tackle are as follow:

(1) The design and optimization complexity is higher than the NRIC due to the multi-coil-based architecture.
(2) The size of the implanted coil is problematic for biomedical applications. Using multiple RX coils (secondary and load coils) increases the real-estate required.
(3) It is challenging to align secondary and load coils inside the human body. This problem is compounded by the fact that the effects of misalignment on the PTE are worse for NRMRC compared to NRIC due to the multi-coil-based architecture.
(4) The parasitic capacitance is usually used to tune the TX and RX coils to their resonating frequency [63]. Additionally, the biological tissue has a higher dielectric constant than the free space [69], which can increase the parasitic capacitance of the implanted coil [137]. Hence, the PTE can be affected by the parasitic capacitance inside the biological tissue.
(5) NRMRC WPT systems have a higher operating frequency than their NRIC counterparts. Therefore, the SAR is prone to the safety limit and must be studied carefully.

The NRMRC has recently been the subject of increased focus compared to NRIC WPT systems due to its higher PTE, larger permissible separation distance and higher bandwidth. Multiple-TX [150,151,152] and RX [153]-based NRMRC WPT systems show great potential for future IMDs.

### 4.6. Verdict

NRMRC demonstrates promising results for IMD applications. It offers better impedance matching, bandwidth and PTE compared to NRIC. However, the multiple coil architecture increases the design and manufacturing complexity. It also can affect the PTE, as this topology is more vulnerable to noise. The lower operating frequency of this technology can also offer acceptable tissue safety. Therefore, this technology can be adopted in the future for the implementation of commercial IMDs.

## 5. Mid-Field WPT

### 5.1. Link Design

Conventional implantable medical devices rely on the non-radiative WPT to support electronic systems. A lower operating frequency range of 100 kHz to 50 MHz reduces tissue losses provided that the WPT systems are of sufficient large size for optimum operation [60]. However, the level of miniaturization required for NRIC and NRMRC implantable systems affects the PTE performance at these low frequencies [13,154], especially when the implant is much smaller than its separation distance from the TX coil [155].

Non-radiative and radiative mid-field (NRRMF) WPT is an emerging technology proposed for powering low power implants deep inside biological tissue where the separation of the TX and RX antenna is of the order of a wavelength from TX antenna. The mid-field WPT scheme for biomedical devices was initially proposed for a 2 × 2 mm^2^ square loop RX implant antenna operating at 915 MHz [13,37]. The received power of NRRMF is, however, significantly lower (<1 mW) than the NRIC and NRMRC schemes [13,156]. In [37,155,157], a combination of non-radiative and radiative modes, known as mid-field WPT, have been utilized to achieve better efficiency at larger distance compared to NRIC and NRMRC for mm-sized implants at sub-gigahertz to the lower-gigahertz range.

**Figure 29:**
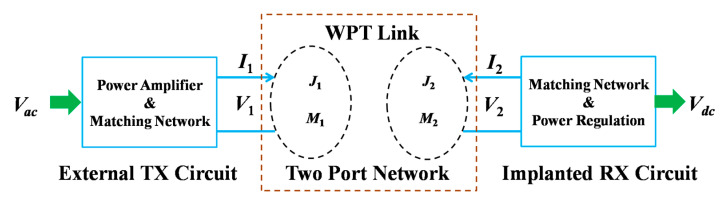
Complete NRRMF WPT system [158]. The input signal is feed to the TX antenna by a power amplifier following by a matching network. The RX antenna is connected with a matching network, and the output signal is processed through a power regulation circuit.

As an example, Figure 29 shows the two-port-based NRRMF WPT system [154,158]. *J*_1_, *M*_1_ and *J*_2_, *M*_2_ are the electric and magnetic current distributions on the TX and implanted RX antennas, respectively. For a mm-sized RX antenna, the TX antenna can be modeled as an infinite sheet of magnetic current density, *M*_1_ [158]. Furthermore, the RX antenna is considered as a combination of magnetic and electric dipoles with arbitrary orientation. The PTE is formulated by abstracting the TX-RX antenna as a two-port network and deriving its performance in terms of coupling and matching efficiency. An optimal operating frequency must be chosen based on the implant depth and type of tissue layers to achieve maximum PTE.

**Figure 30:**
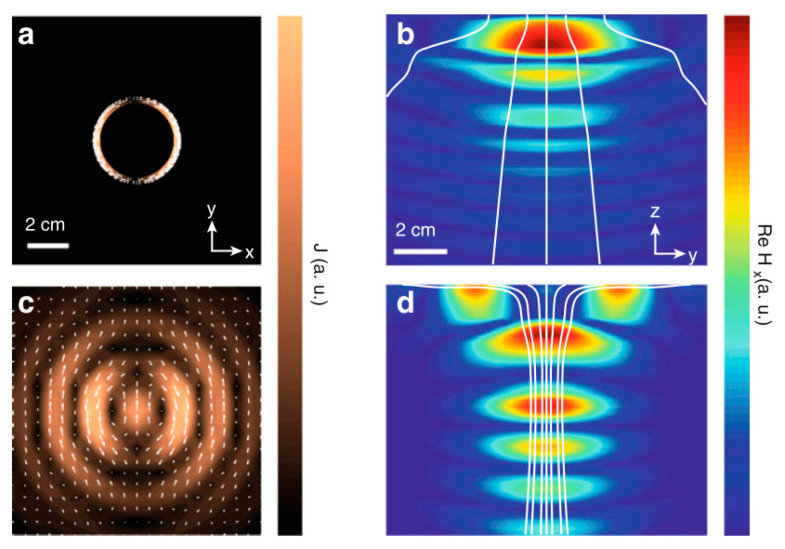
Current density and magnetic field distribution for a coil source (**a**,**b**) and optimal source (**c**,**d**) at 2.6 GHz [48,154]. The magnetic field component aligned with the receiver dipole moment and the Poynting vector (white lines) generated by the coil source and the optimal source. Copyright © 2013, IEEE.

In Figure 30, the current density and generated magnetic fields are compared for a traditional coil source (NRIC) as shown in Figure 30a,b and an optimal NRRMF source, Figure 30c,d [48,154]. The effect of an optimal source results in a focusing effect of the magnetic field component and power flow, as shown in Figure 30d, which is responsible for higher PTE.

### 5.2. Optimization

The PTE of a two-port network shown in Figure 29 for a mm-sized and loosely coupled RX antenna is written as [158]:

(34) $$\eta =\frac{{\left|Z_{21}\right|}^{2}}{4R_{11}R_{22}}\frac{4R_{22}R_{L}}{{\left|Z_{22}+Z_{L}\right|}^{2}}=\eta_{c}\eta_{m}$$

where *Z_mn_* and *Z_L_* are the network and load impedance parameters, respectively, of the two-port network, and *R*_11_ and *R*_22_ are the real parts of *Z*_11_ and *Z*_22_. This PTE is the product of the coupling efficiency, *η_c_* (first term on the R.H.S of the equation) and the matching efficiency, *η_m_* (second term). The maximum PTE is independent of the antenna structure and relies solely on the maximization of the coupling parameter *γ* [158]:

(35) $$\gamma =\frac{{\left|Z_{21}\right|}^{2}}{R_{11}}$$

This parameter decreases as the power loss due to *R*_11_ increases. It is also an increasing function of the frequency although this increase must be traded off against larger tissue dielectric losses. After incorporating the tissue losses, the optimal frequency is calculated by considering only the lowest order multipole as:

(36) $$f_{opt}=\frac{1}{2\pi }\sqrt{\frac{c\sqrt{\epsilon_{r0}}}{\tau d\left(\epsilon_{r0}-\epsilon_{\infty }\right)}}$$

where *τ* is the tissue relaxation time constant, *c* is the speed of light and *d* is the separation distance. *ϵ_ro_* and *ϵ_∞_* are the relative permittivity values of the tissue at dc and infinite frequencies and can be found in [159].

### 5.3. System Design

**Figure 31:**
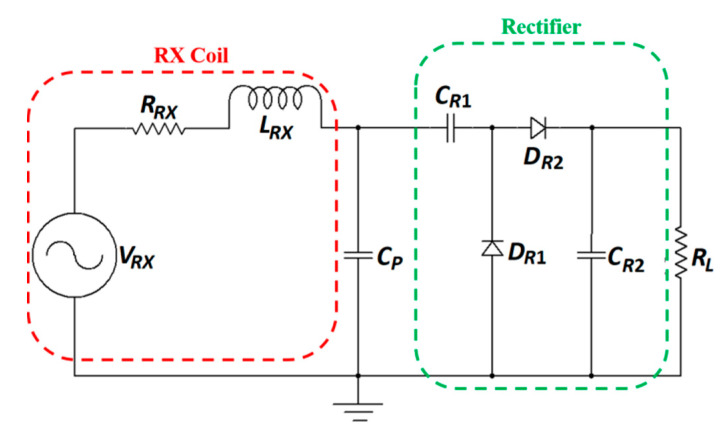
RX circuit for NRRMFWPT system.

The designs of the power amplifier and matching network design are rarely discussed in literature for NRRMF WPT. A high-efficiency amplifier is challenging to design in the GHz frequency range mainly due to the influence of parasitics. Researchers mostly utilize the two-port network analyzer to test and validate NRRMF WPT technique. Figure 31 shows an example of a RX circuit for NRRMF at 1.4 GHz [154]. It consists of an RX coil, an impedance matching capacitor, *C_p_*, a single-stage full-wave rectifier and a dc load, *R_L_*. *D_R1_* and *D_R2_* are the Schottky diodes. The RX coil is modeled as an equivalent voltage source, *V_RX_* with inductance, *L_RX_* and self-resistance, *R_RX_*. A matching circuit (Figure 29) is designed based on the impedance of the RX coil, but is independent of the impedance of the rectification circuit. The design of the rectifier depends on the voltage and current requirement from the load. An ASIC-based rectifier is suggested in [154] due to its low footprint and higher efficiency compared to Schottky diode-based rectifiers.

### 5.4. Applications

#### 5.4.1. CardiacImplant

**Figure 32:**
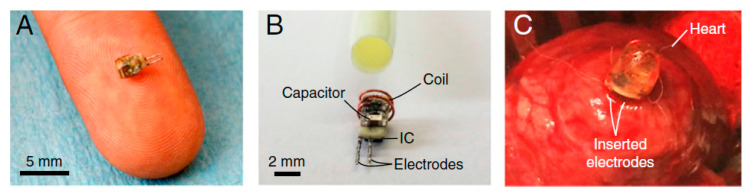
Wireless cardiac pacing implant [156]. (**A**) The wireless electrostimulator of 2 mm in diameter. (**B**) The samedevice before epoxy encapsulation next to a 10-French (~3.3mm) catheter sheath for size comparison. (**C**) The electrostimulator inserted in the lower epicardium of a rabbit via open-chest surgery. Reproduced with permission of PNAS.

Figure 32 shows a cardiac implant application [156]. The wireless electro-stimulator of 2 mm diameter, approximately 4 mm in length and 70 mg of weight, was inserted into the lower epicardium of a rabbit. The heart rate of the rabbit was monitored by Electrocardiogram ECG. The separation distance of the TX and RX antenna is approximately 5 cm. For a 500 mW transmitted coupling power, 200 μW of RX power leading to a PTE of 0.04% and a maximum SAR of 0.89 W/kg was achieved.

#### 5.4.2. Neurostimulator Implant

**Figure 33:**
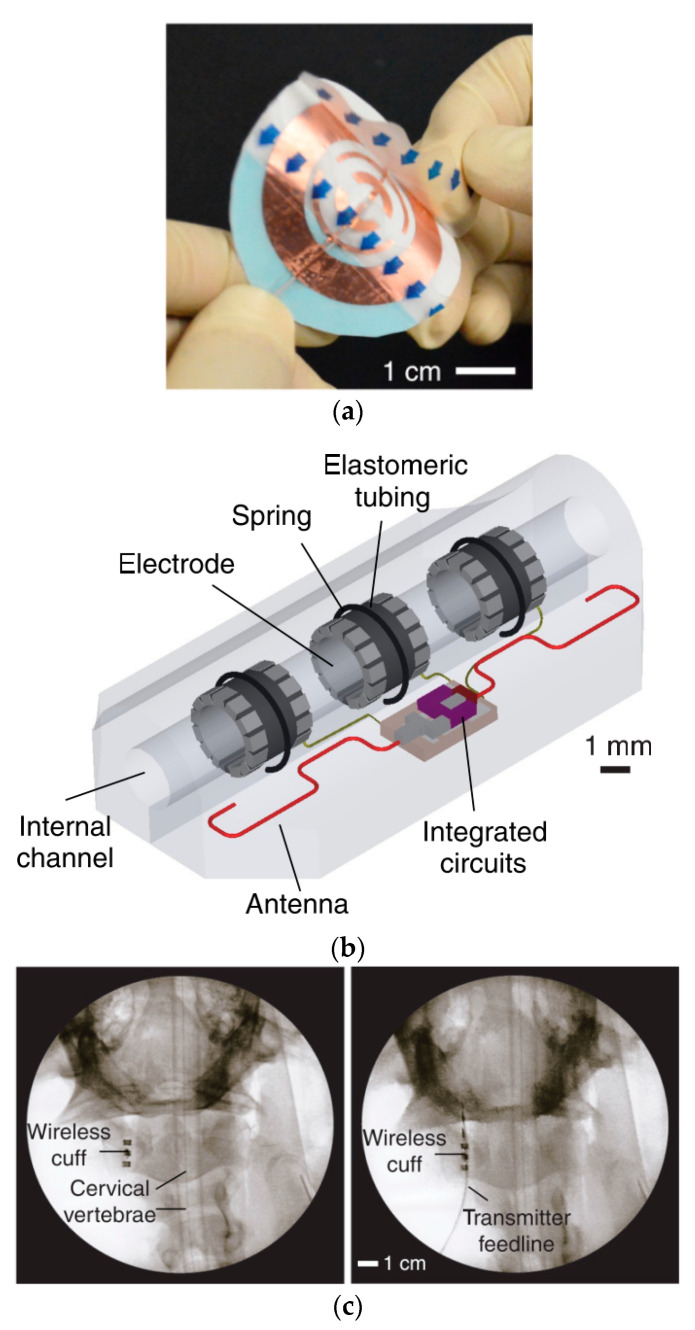
Wireless peripheral nerve neuromodulation system [160]. (**a**) Conformal wireless powering transmitter. (**b**) Design of wireless cuff electrodes. (**c**) Fluoroscopy images of the implanted wireless cuff and transmitter on skin. Copyright © 2017 Tanabe et al.

Figure 33a shows the design of a WPT conformal transmitter consisting of reactively loaded rings laser-cut from copper film and encapsulated in soft silicon [160]. The outer diameter of the transmitter is 30 mm. A dipole antenna is used to extract the wireless power for the neuromodulation device, as shown in Figure 33b. The frequency of the proposed system is 2.4 GHz. For the transmitter placed on the human neck above the vagus nerve, the maximum TX power is measured at 180 mW. The maximum receiver PTE is claimed to be more than 20% for 1 kΩ load, but decreases to 13% for a 150 Ω load. The cuff device was attached to the right cervical vagus nerve of anaesthetized pigs, as shown in Figure 33c. The transmitting antenna is attached to the skin surface approximately 15 mm above the implanted device. The simulated SAR is recorded as 2 W/kg for 10 g of tissue.

#### 5.4.3. Capsule Endoscopy

**Figure 34:**
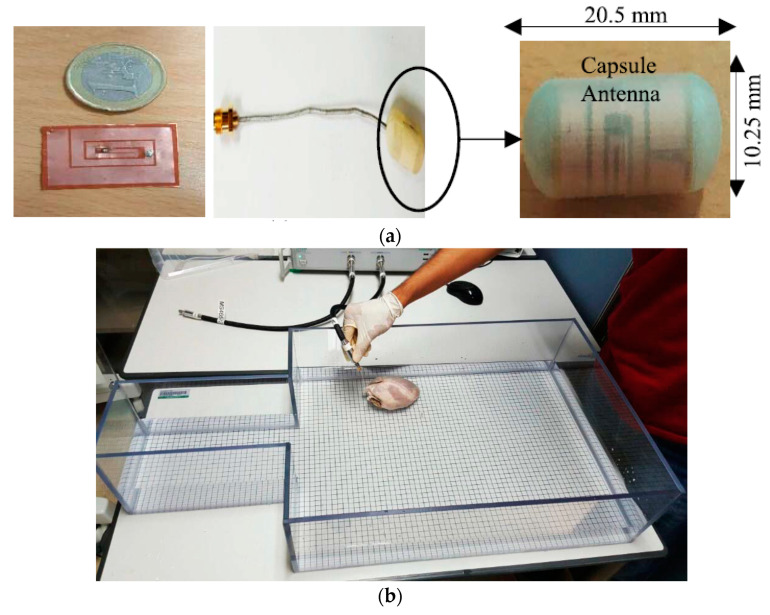
NRRMF WPT system for CE [161]. (**a**) Fabricated conformal antenna and 3D capsule prototype. (**b**) Measurement setup in ASTM phantom. Copyright © 2017, IEEE.

In [161], a multi-band conformal antenna is presented for 402–405 MHz and lower GHz frequency for CE applications. Figure 34a shows the fabricated conformal antenna printed on a cylindrical capsule of 10.25 mm outer diameter and 20.5 mm length. A two-port TX coil was designed for power transmission [156]. The proposed model was tested in an American Society for Testing and Materials (ASTM) phantom containing a porcine heart and saline water, as shown in Figure 34b. For a 5 mm separation distance inside porcine heart the received power is reported as 800 μW for 1 W of TX power. No SAR measurement was reported.

### 5.5. Design Challenges and Future Trends

The NRRMF WPT scheme is a potential solution for deep implant mm-sized RX. However, some challenges have to be met before utilizing this technique for biomedical applications.

(1) The output power of NRRMF WPT systems is very low, which limits the range of applications for this method. The lower PTE limits the output power due to the higher separation distance in NRRMF. It is possible to increase the output power by improving the TX power within tissue safety guidelines.
(2) The PTE in this technique is maximized by focusing the TX power towards the RX. Hence, the influence of misalignment on the PTE could be significant and needs to be studied carefully before considering this technology.
(3) The design of high-efficiency amplifier and rectifier at sub-GHz and GHz frequency can be a challenging task.
(4) Tissue safety is the major issue of concern due to the higher frequency region selected for this method.

The ability of NRRMF WPT systems to focus energy makes it an attractive proposition for the treatment of tumors and cancer cells.

### 5.6. Verdict

NRRMF offers a better prospect for the miniaturization of the WPT coils compared to NRIC and NRMRC, which is one of the key design specifications for IMDs. However, the RX power is significantly lower to support multi-modal IMDs. Furthermore, the higher MHz and sub-GHz operation frequency range can lead to severe tissue damage for more extended usage period in a patient. The system-level design complexity is also higher at the operation frequency range of NRRMF-based WPT techniques. Furthermore, systematic design and optimization are still absent in the literature for this WPT technique.

## 6. Radiative Far-Field

### 6.1. Link Design

Radiative far-field (RFF) WPT relies on the electromagnetic coupling of an RX antenna positioned at a large separation distance (*d* >> *λ*) from the TX antenna. Compared with NRIC and NRMRC, this transfer technique demonstrates robustness against misalignment of the TX and RX coils [162,163]. RFF WPT has been studied extensively over the last decade in free-space [164,165,166,167]. On the other hand, the study of this technology for biomedical implants is still in its early stage [168,169].

**Figure 35:**
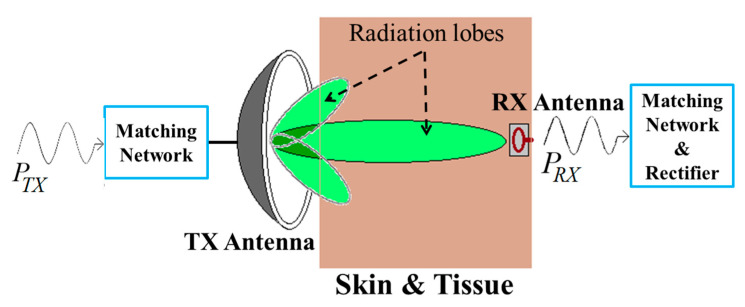
RFF WPT system powered by alternative EMF.

The tissue safety concerns regarding the RFF WPT system are illustrated in [162,169,170]. Figure 35 shows an RFF WPT system to deliver EM radiation toward the RX antenna from a TX antenna at an optimal frequency [164,167]. The parameters of the TX and RX antennas have been optimized in free space and in a human body model present in the finite field electromagnetic (EM) solver software, such as CST microwave studio and ANSYS HFSS [162,169,170]. The Federal Communications Commission (FCC) regulated a maximum TX output power as 30 dBm or 1 Watt [171]. Furthermore, the maximum isotropic radiated power (EIRP) should be less than 36 dBm or 4 Watts [162,169]. Additionally, the maximum permissible exposure (MPE) for the uncontrolled exposure to an intentional radiator is limited to 6 and 10 W/m^2^ for operating frequencies of 915 MHz and 2.4 and 5.8 GHz, respectively [162]. Hence, the maximum transfer distance can be calculated as 0.178 m for the maximum limit of EIRP and MPE for an RFF WPT system.

### 6.2. Optimization

In the far-field, the radiated fields can be modeled as plane waves with electric and magnetic field components as *E_θ_* and *H_φ_*, respectively. The simplified expression of the power radiated by the TX antenna is [172]:

(37) $$P_{TX}=\int_{0}^{2\pi }\int_{0}^{\pi }U\sin\theta d\theta d\varphi$$

where *U* is the radiation intensity of the far-zone electric field given as [172]:

(38) $$U=\frac{r^{2}}{2\eta }\left[{\left|E_{\theta }\right|}^{2}+{\left|E_{\varphi }\right|}^{2}\right]$$

where *η* and *r* is the intrinsic impedance of the medium and closed surface radius of the radiation boundary of the TX antenna, respectively [172]. This radiated field is incident on the matched RX antenna and generates a current across the RX antenna terminals. The received power, *P_RX_*, can be calculated using Friis radio link formula as [169]:

(39) $$P_{RX}=\frac{G_{TX}G_{RX}\lambda_{o}^{2}}{2\pi d}\left(1-{\left|S_{11}\right|}^{2}\right)\left(1-{\left|S_{22}\right|}^{2}\right)e_{p}\times P_{TX}$$

where *G_TX_* and *P_TX_* are the gain and transmitted power of the TX antenna, respectively, and *S*_11_ and *S_22_* are some of the scattering parameters of the RX antenna. *G_RX_* is the gain of the RX antenna and *e_p_* is the polarization mismatch of antennas [173].

The first physical limitation in terms of Q-factor for small antennas bounded by a sphere was given by Chu in the 1940s [174]. The Q-factor is of primary importance in the design and analysis of electrically small antennas because of its approximate proportionality to the fractional bandwidth (*BW*) [175,176]. For a low Q-factor, the input impedance of the antenna varies slowly with the frequency and demonstrates broad bandwidth. This lower bound Q-factor, *Q_lb_*, is quantified as [177,178,179]:

(40) $$Q_{lb}=\frac{1}{{\left(ka\right)}^{3}}+\frac{1}{ka}≅\frac{1}{BW}$$

where *k* = 2π/*λ* is the free-space wave number and *a* is the radius of the imaginary sphere circumscribing the electrically small dipole antenna. The *Q_lb_* introduces a fundamental limitation on the bandwidth-efficiency product of a small resonance type antenna [180]. Therefore, when the implantable RX antenna size is made smaller, the bandwidth will increase in such a way that the bandwidth-efficiency product remains constant hence reducing the radiation efficiency.

The end to end power transfer efficiency of the RFF WPT system can be expressed as [167]:

(41) $$\eta_{e2e}≜\frac{P_{OUT}}{P_{IN}}=\eta_{1}\eta_{2}\eta_{3}\eta_{4}\eta_{5}\;=\frac{P_{TX}}{P_{IN}}\times \frac{P_{ERP}}{P_{TX}}\times \frac{P_{INC}}{P_{ERP}}\times \frac{P_{RX}}{P_{INC}}\times \frac{P_{OUT}}{P_{RX}}$$

where *P_OUT_* and *P_IN_* denote the input and output dc powers of the complete system. Moreover, *P_TX_*, *P_ERP_*, *P_INC_* and *P_RX_* are defined as the TX power, effective radiated power, incident power and RX power, respectively. Despite the significant improvement in the transmission efficiency, *η*_1_ × *η*_2_, and reception efficiency, *η*_4_ × *η*_5_, the free-space propagation efficiency, *η*_3_, has the most substantial influence on *η*_e2e_.

### 6.3. System Design

**Figure 36:**
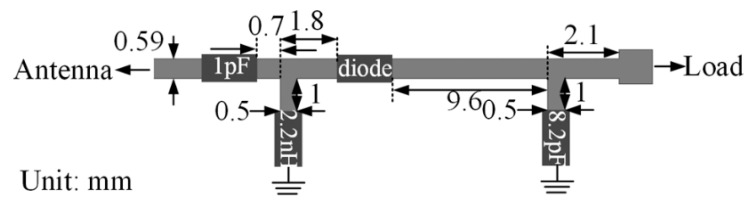
Rectifier schematic for RFF [169]. Copyright © 2014, IEEE.

Design of a high-efficiency power amplifier is rare in the literature. Therefore, researchers utilize a two-port network analyzer to test such WPT link. Figure 36 shows the example of a rectifier for RFF WPT for IMDs [169]. The Schottky diode, HSMS-2852, is used to rectify the RFF RX power at 2.45 GHz frequency. The source impedance of the rectifier is set at 50 Ω to match the impedance of the RX antenna directly without any impedance mismatch.

**Figure 37:**
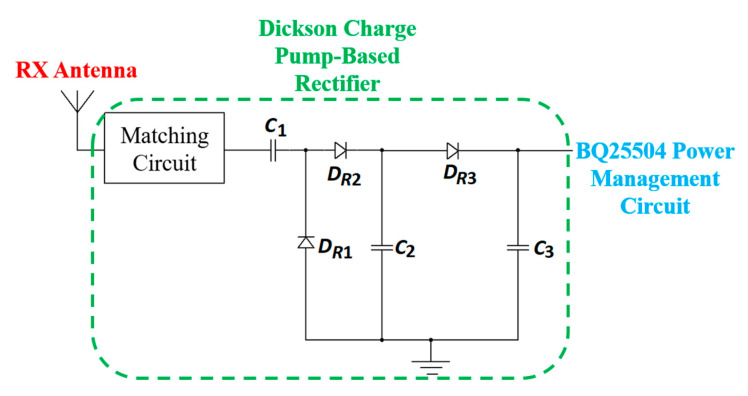
RX power processing unit for RFF [165].

In [165], a single-stage Dickson charge pump is used as a rectifier. This charge pump works as a voltage multiplier circuit. In the second stage, an ultra-low power DC-DC converter, BQ25504, is used to boost the output of the first stage rectified output, as shown in Figure 37.

A methodology is proposed in [164] to determine the optimal impedance of the rectifier in order to accurately design and measure the output of the rectifier circuit for RFF. In [181], a CMOS TI 130 nm technology is utilized to manufacture p-type Schottky diode-based rectifier for RFF WPT inside a proposed IMD ASIC.

### 6.4. Applications

**Figure 38:**
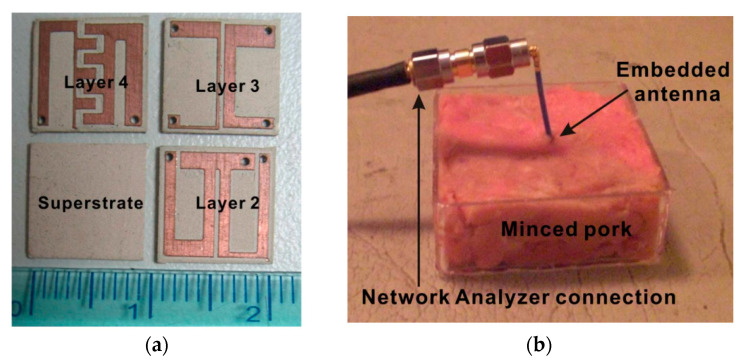
RFF WPT antenna [183]. (**a**) Fabricated triple-band miniaturized antenna. (**b**) Measurement setup embedded in minced pork. Copyright © 2011, IEEE.

A few attempts have been recorded in recent years regarding the implementation of an RFF WPT system for IMDs [181,182,183,184,185,186,187] Published studies are rare where an RFF WPT antenna is tested thoroughly inside biological tissues. In [182], a broadband monopole antenna using a general substrate is presented for the medical implant communication service (MICS) frequency band of 402~405 MHz. The size of the receiver antenna is 18 × 16 mm^2^. The maximum SAR observed is 0.02 W/kg for 1 g tissue, and 25 μW of power is delivered to the implantable antenna. A triple-band biotelemetry antenna with data telemetry (402 MHz), WPT (433 MHz) and wake-up control (2.45 GHz) is reported in [183]. Figure 38a shows the triple-band fabricated antenna of size 10 × 10 × 2.54 mm^3^. The proposed antenna was tested in a 65 × 92 × 50 mm^3^ minced pork, as shown in Figure 38b. The peak SAR measured was 1.6 W/kg for 1 g of tissue and 5 mW of input power. The PTE is reported as 15%.

**Figure 39:**
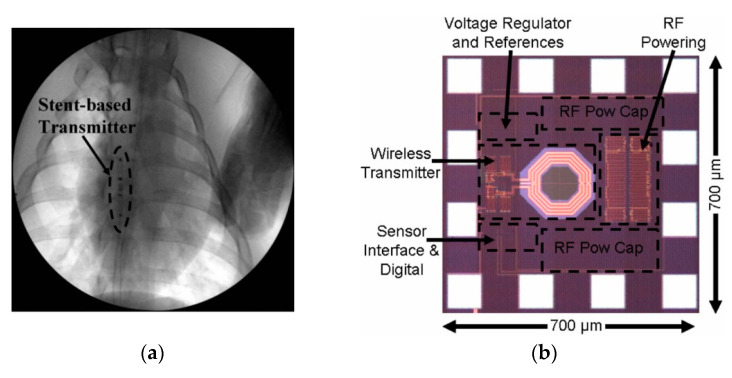
Implantable cardiovascular pressure monitoring system [181]. (**a**) Radiograph of stent implanted in the chest cavity of a live porcine. (**b**) Optical microscope picture of ASIC including WPT antenna. Copyright © 2010, IEEE.

A single-fed hybrid patch/slot antenna for MICS band is presented in [184]. The maximum SAR is 2 W/kg for 10 g of tissue and 20.5 mW input power. A 10.02 × 10.02 × 0.675 mm^3^ dual-band antenna is presented for MICS and industrial, scientific and medical (ISM) bands [185]. The maximum SAR is 1.6 W/kg for 1 g of tissue and 5.3 mW of input power. In [181], a medical stent with a fully wireless implantable cardiac pressure monitor system is presented. Figure 39a shows the in vivo experiment of the proposed cardiac pressure monitoring transmitter inside the chest cavity of an anaesthetized live pig. The ASIC, shown in Figure 39b, is directly connected to the end of the stent and includes the wireless transmitter, WPT antenna and power processing units. The power is transferred at 3.7 GHz frequency at 100 mm distance. For the input power of 3.6 W, the proposed system can generate 2.5 V of rectified voltage, which is necessary for the reliable performance of this system. The average simulated SAR is claimed as 2.29 W/kg. No measurement of the PTE is provided.

### 6.5. Design Challenges and Future Trends

In the RFF WPT scheme, the overall PTE is inherently low, and several design challenges have to be resolved to improve the efficiency alongside tissue safety considerations.

(1) One of the significant limitations of RFF powering in free space is the power density decrease as 1/*d*^2^ due to energy spreading [162]. In the case of implantable devices, the power density attenuation increases significantly as the EM waves pass through biological tissue.
(2) To satisfy the safety regulations set by the FDA and FCC for far-field based WPT systems, the radiated TX power and received power at the implant side are small compared to the NRIC and NRMRC WPT systems.
(3) The design complexity of the TX and RX circuits increases because of the higher operating frequency of the RFF WPT system.

RFF WPT systems have also emerged that have field beam-steering antenna capability to charge a mobile phone. Such capability could be applied for the localization of IMDS [176,188,189,190]. The design of TX and RX circuits for higher frequency RFF application is also an important future research topic.

### 6.6. Verdict

RFF is a WPT technique rarely studied in the literature for IMD applications. The high operating frequency makes the overall system implementation complex. Furthermore, the tissue safety requirement limits the amount of power transferred by this technique. Therefore, it is inappropriate for most of the multi-modal IMDs.

## 7. Acoustic Power Transfer

### 7.1. Link Design

Acoustic power transfer (APT), a non-EM WPT technique, is the leading competitor against NRIC and NRMRC in terms of PTE performance [191,192]. In the APT technique, energy is wirelessly propagated using ultrasound waves at carrier frequencies above 20 kHz without interfering with EM waves [193]. APT uses a pair of piezoelectric transducers to transfer energy in the form of ultrasound waves through tissue to an implanted device where it is converted to electric power.

**Figure 40:**
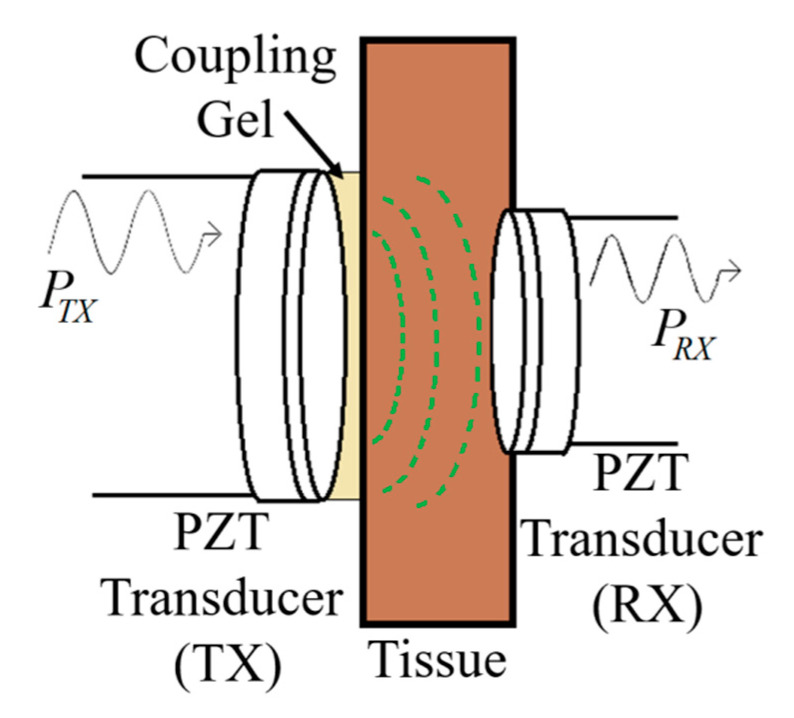
Typical acoustic power transfer (APT) link.

Figure 40 shows a typical APT system where TX is an ultrasonic oscillator. It is excited electrically to produce surface vibrations resulting in acoustic pressure waves in the frequency range of 200 kHz to 1.2 MHz. The pressure field is to be directed towards the RX transducer, and the directivity depends on the ratio of the transducer perimeter to the wavelength. Arrays of transducers can also be implemented for better directivity at the costs of reduced beam penetration. A Gaussian shading-based ultrasonic transcutaneous energy transfer (UTET) transmitter for 650 kHz continuous wave is presented in [194]. Better and safer performance over a Bessel or uniform excitation is demonstrated by reducing the pressure variation and spreading of the power flow over the cross-sectional area. The RX transducer is implanted inside the body to harvest piezoelectric energy and is positioned within the main radiation lobe of the TX to convert back the acoustic energy to electrical energy [18,195].

### 7.2. Optimization

**Figure 41:**
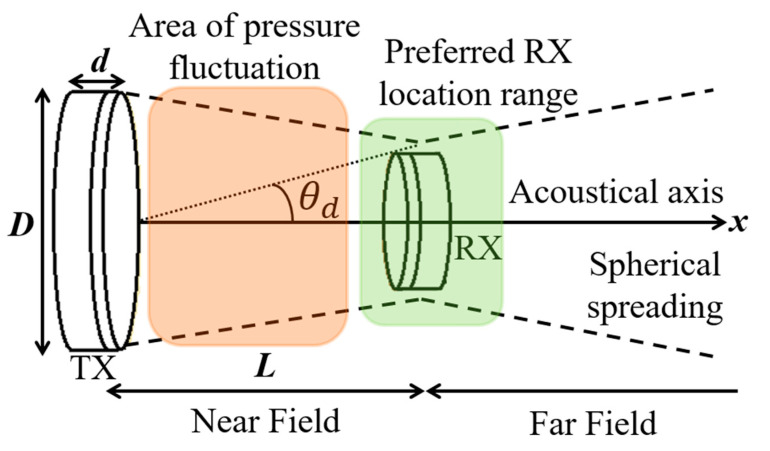
Representation of near-field and far-field regions of an acoustic wave generated by TX and incident on RX.

APT for IMDs depends on the generation and propagation of sound through the biological medium. The finite speed of the acoustic wave propagation is a function of the elastic properties and density of that medium. The behavior of the acoustic wave can be separated into two regions: the near-field and far-field, as shown in Figure 41. The near field is the closest to the TX transducer, and the pressure field envelope oscillates in this zone resulting in several minima and maxima of power. The far-field is a smooth spherically spreading wave decaying with increasing distance. In the region corresponding to the transition between near and far fields, the beam waist of the acoustic beam is at its smallest. This region defines the preferred location where RX should be installed. This transition distance, defined as the distance between TX and this region and also known as Rayleigh distance, can be defined as [18] in Equation (42).

(42) $$L=\frac{\left(D^{2}-\lambda^{2}\right)}{4\lambda }\approx \frac{D^{2}}{4\lambda },D^{2}\gg \lambda^{2}$$

where *D* is the aperture width of TX and *λ* is the wavelength of the acoustic wave in the medium. At the Rayleigh distance, the acoustic beam spreads out at divergence angle given as:

(43) $$\theta_{d}=\sin^{-1}\left(\frac{1.22\lambda }{D}\right)$$

The maximum achievable received power occurs at the Rayleigh distance where the acoustic pressure is maximum and relatively constant [196]. The position of the maxima of pressure along the acoustic axis in the near field region can be approximated as:

(44) $$X_{max}\left(m\right)=\frac{D^{2}-\lambda^{2}{\left(2m+1\right)}^{2}}{4\lambda \left(2m+1\right)}$$

where *m* (=1, 2, 3…) is the order of the pressure peaks.

An improper selection of the transmission frequency can adversely impact on the tissue attenuation, Rayleigh distance and sizes of the TX and RX transducers. The maximum power that can be delivered by a transducer is achieved at its resonant frequency, *f_r_*, which depends on the geometry and material of the transducer [197] so that:

(45) $$f_{r}=\frac{c}{2d}$$

where *c* is the acoustic velocity in the piezoelectric transducer and *d* is its thickness, as shown in Figure 41. Increasing *f_r_* helps reduce the transducer size and to increase the Rayleigh distance at the cost of increased tissue absorption.

The PTE of the APT link typically depends on transducer losses, the amount of power tapped by the RX, acoustic impedance matching layer losses, losses due to tissue absorption and rectifier losses. The PTE for the link is defined as [197]:

(46) $$\eta =\left|\frac{P_{OUT}}{P_{IN}}\right|=\frac{{\left(\mu TNR_{L}\right)}^{2}}{C_{TX}C_{RX}V_{IN}^{2}{\left(R_{L}+Z_{OUT}\right)}^{2}}$$

where *C_RX_* is the capacitance of the receiver, *Z_OUT_* (=1/*jωC_RX_*) is the output impedance; *μ* = *e*^−2*αx*^ is the tissue attenuation factor, where *α* is the attenuation coefficient and *x* is the depth of the implant; *N* is the turn ratio of the electrically equivalent model of a piezoelectric transducer [198] and *T* ≈ 2|*Z_receiver_*| × *c. Z_receiver_* is the acoustic impedance of the receiver defined as the product of its density and the speed of sound in the material [199].

### 7.3. System Design

**Figure 42:**
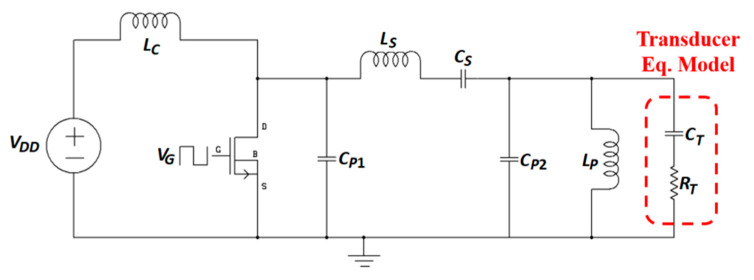
Shunt-C class-E amplifier.

Figure 42 shows a shunt-C class-E amplifier driving an electrically equivalent model of a TX transducer *C_T_*, *R_T_* [200,201]. The parallel inductor, *L_P_* resonates at 1 MHz frequency with the transducer. The series capacitor, *C_S,_* is used to prevent dc feed through. The parallel capacitors, *C_P_*_1_ and *C_P_*_2_ confirm the nominal condition of the amplifier. Furthermore, the series inductor, *L_S_* is utilized to improve the efficiency of the amplifier. The amplifier maximum efficiency achieved as 71%.

**Figure 43:**
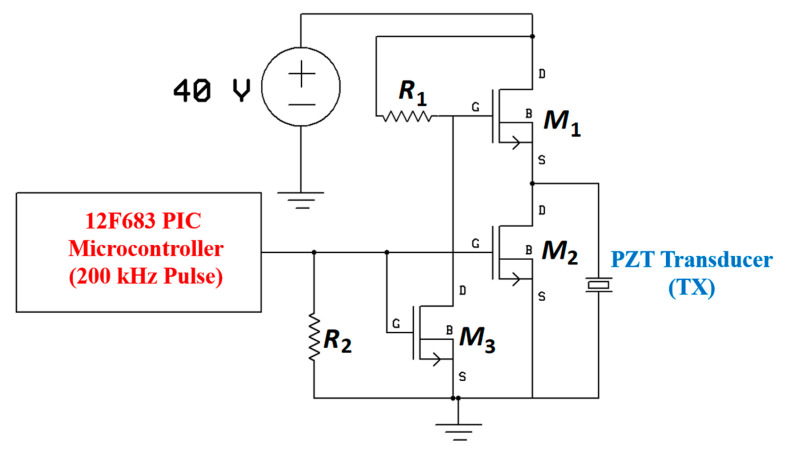
Schematic ofthe piezoelectric (PZT) driver.

An alternative driving circuit for TX piezoelectric (Lead ZirconateTitanate - PZT) transducer is proposed in [193,202] using the 12F683 Microchip PIC microcontroller and 2N7000 NMOS transistors. The driver generates 200 kHz pulses with an input supply of 40 V, as shown in Figure 43. The driver signal with a 43% duty ratio is fed to the gates of the *M*_2_ and *M*_3_ transistors to switch the input supply of 40 V across the TX PZT disc. During start-up, the totem-pole configuration on the transistors creates an initial potential equal to the supply voltage across the TX via *M*_1_. The positive pulse from the microcontroller is responsible for turning *M*_2_ and *M*_3_ on while *M*_1_ is off supplies a low output to the TX. During the negative pulse, the opposite operation transfers higher voltage to the TX.

An active rectifier topology of 83% efficiency is presented in [201] for the RX power processing of an APT system. Other conventional bridge rectifiers are presented in [203,204]. A recent attempt [205] demonstrated an ASIC-based active rectifier and regular for the APT link.

### 7.4. Applications

Different applications of APT for biomedical implants found in the last decade are described in the following sections.

#### 7.4.1. Micro-Oxygen Generator

In [206], an ultrasonically powered implantable micro-oxygen generator (IMOG) is presented that is capable of in situ tumor oxygenation through water electrolysis. Ultrasound is used to enable deep implantation of the IMD. The frequency of the APT was 2.15 MHz.

**Figure 44:**
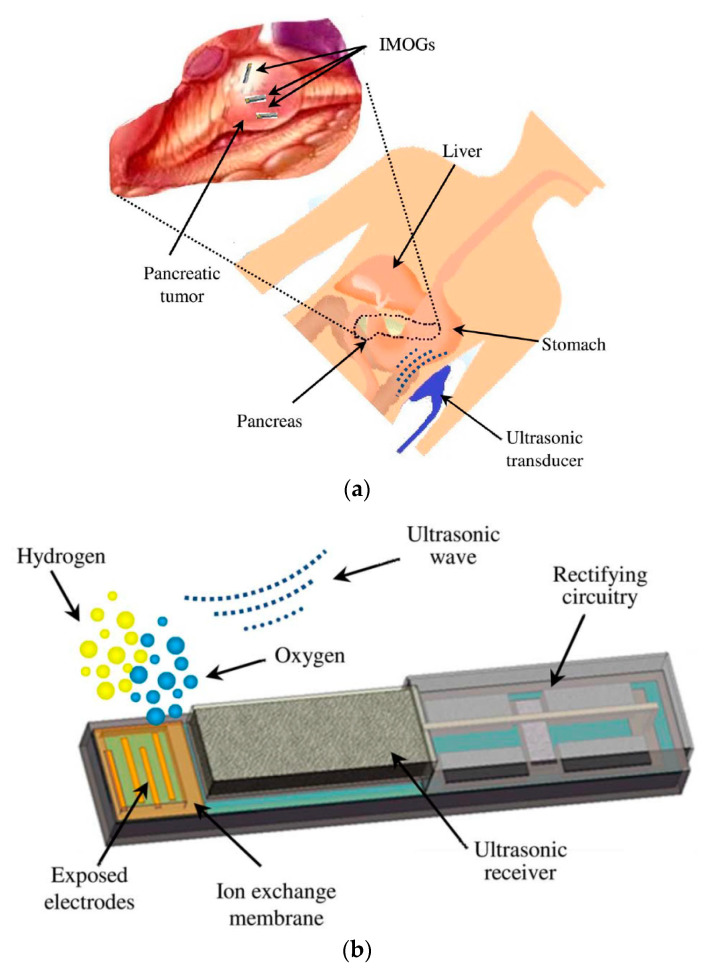
Proposed implantable micro-oxygen generator (IMOG) device [206]. (**a**) Conceptual view of IMOG implanted in a pancreatic tumor. (**b**) Various components in a complete IMOG including ultrasonic power RX. Copyright © 2011, IEEE.

**Figure 45:**
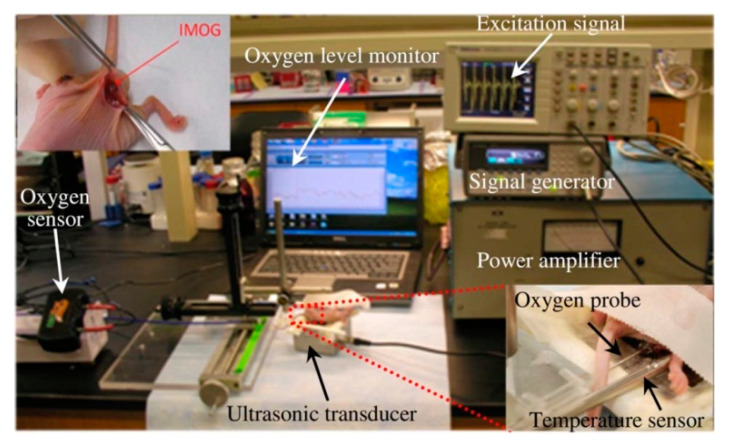
Experimental setup for real-time in vivo oxygen measurement during tumor oxygenation by IMOG [206]. Copyright © 2011, IEEE.

Figure 44a shows the conceptual design of the IMOG to be located inside a pancreatic tumor and powered ultrasonically from the outside. Once energized, the proposed device can perform in situ electrolysis by utilizing the water present in the tissue to generate oxygen. Figure 44b provides the complete schematic of the device of dimensions of 1.2 × 1.3 × 8 mm^3^. The in vivo experiment of the proposed IMOG system is shown in Figure 45 for a pancreatic tumor model grown in the flanks of the athymic mice. The wireless power recorded is 330 μW at a separation distance of 30 mm. Measurement of the SAR of the tissue is not reported.

#### 7.4.2. Bladder Pressure Sensing

**Figure 46:**
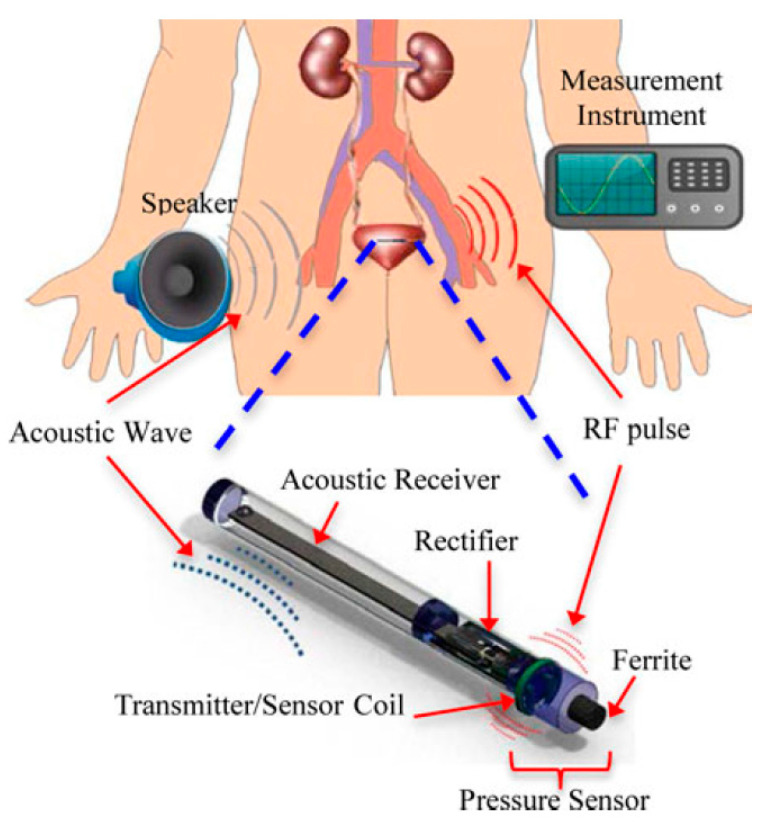
Schematic of APT LC transponder implanted in the bladder [203]. Copyright © 2014, IEEE.

**Figure 47:**
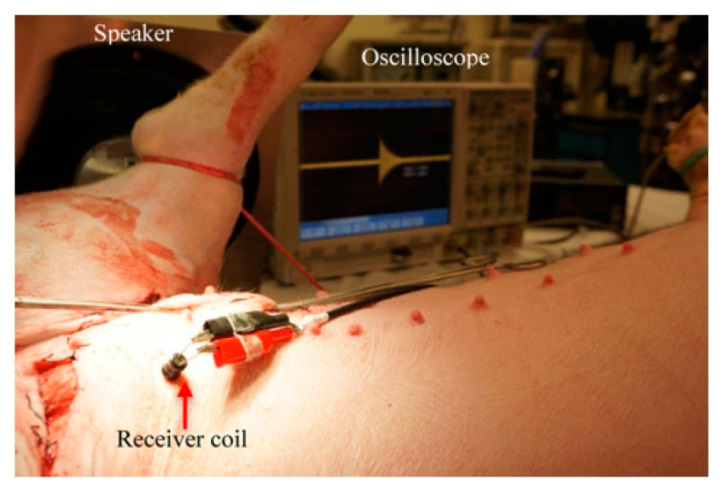
Photograph of in vivo experiment inside pig’s bladder [203].

Figure 46 shows an implantable LC pressure sensing system for the bladder that is powered by APT at 350 Hz [203]. The RX piezoelectric (PZT) cantilever, of 20 × 2 × 0.38 mm^3^ dimensions, is excited by audible acoustic waves from a speaker. The maximum output power is 16 μW for a conversion efficiency of 1.4 × 10^−4^% at 100 mm separation distance. In vivo experiment of the proposed model, as shown in Figure 47, was demonstrated, but tissue safety analysis is unavailable for the proposed model.

#### 7.4.3. Localized Photodynamic Therapy

**Figure 48:**
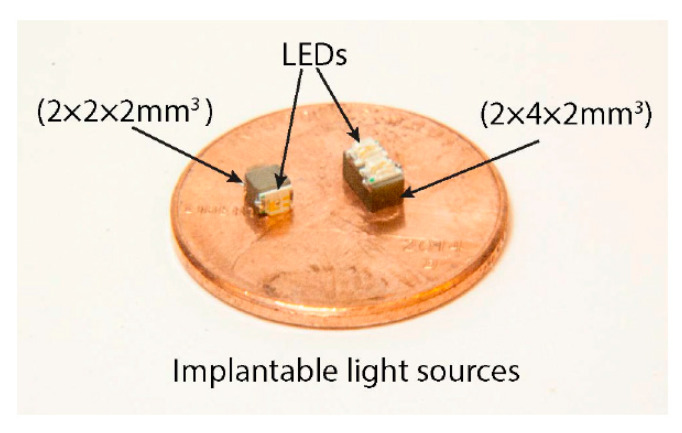
Mounted micro-light sources on PZT cubes on a US penny. One LED, and two LEDs are mounted on each side of 2 × 2 × 2 mm^3^ and 2 × 4 × 2 mm^3^ PZT, respectively [207]. Copyright © 2015, IEEE.

**Figure 49:**
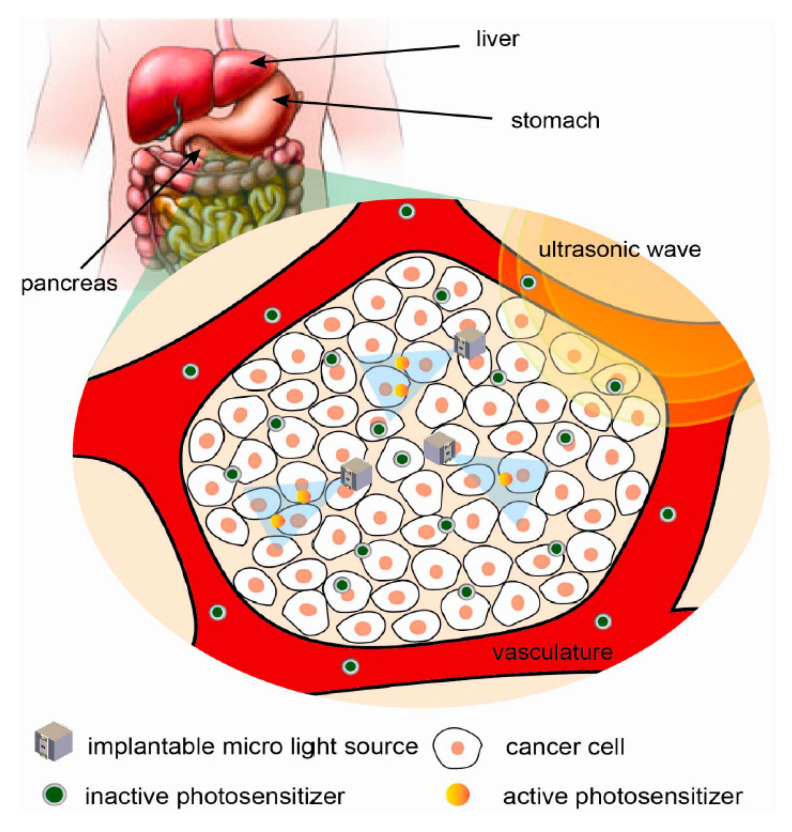
Proposed system showing several implantable micro-light sources to activate the photosensitizer deep inside a tumor powered by ultrasonic wave [207]. Copyright © 2015, IEEE.

In situ localized light for photodynamic therapy using an ultrasonically powered light source is presented in [207]. The implants are 2 × 2 × 2 mm^3^ and 2 × 4 × 2 mm^3^ in dimensions and consist of two red LEDs mounted on a PZT energy source, as shown in Figure 48. The proposed ultrasonically powered implantable micro-light source, shown in Figure 49, is to deliver light deep inside a tumor. An ultrasonic wave of 672 kHz strikes the PZT receiver built inside the micro-light to generate electrical power to turn the on-board LEDs on. The light from the LEDs activates the pre-delivered photosensitizer to initiate photodynamic therapy. The proposed system is tested in 10 mm thick porcine tissue. The achieved output power, efficiency and tissue safety analysis is not reported in this research.

#### 7.4.4. Electrical Stimulation of Peripheral Nerves

**Figure 50:**
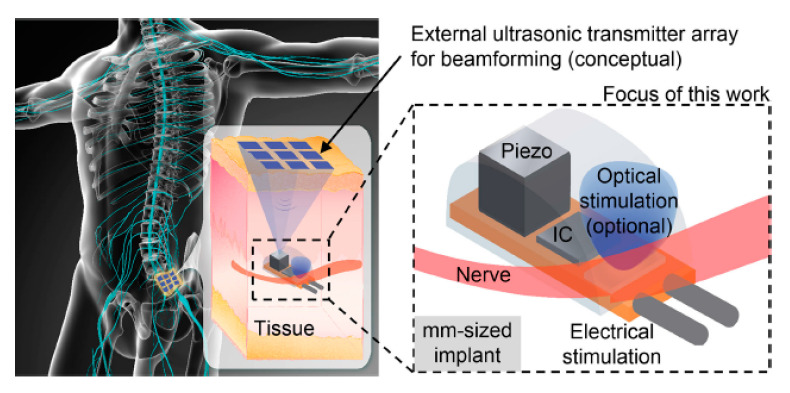
Conceptual diagram of the proposed electrical stimulation implant [205]. Copyright © 2018, IEEE.

A wireless electrical implant, of 2 × 3 × 6.5 mm^3^ dimensions, to stimulate peripheral nerves was manufactured using PZT receiver is presented [205]. Figure 50 shows the conceptual model of the proposed system. An external TX beams the ultrasonic power and downlink data to the implant to support fully programmable stimulation of a peripheral nerve.

The frequency of the RX is 1.314 MHz. The available received power is reported as 3 mW using a commercial TX transducer for a separation distance of 10.5 cm. The proposed model was tested for frog static nerve stimulation. SAR value inside the tissue is not reported.

### 7.5. Design Challenges and Future Trends

Besides NRIC and NRMRC WPT techniques, APT is a strong contender for WPT of biomedical implants. However, reports of proper link modeling and characterization of the effects of ultrasound wave inside the human body are scarce compared to the EM WPT techniques [157,208]. Therefore, the following design challenges have to be resolved before utilizing the APT system in implantable devices.

(1) One of the significant challenges associated with designing an APT system is the different density and acoustic impedance of different organs in the human body. The acoustic impedance of bones is high enough to reflect all ultrasound waves. Attenuation of sound by the soft tissue layers increases exponentially with increasing frequency and distance [209,210]. Therefore, APT is limited to specific body parts for powering implanted devices. Continuous tissue vibration is another health concern for APT systems [211].
(2) For deep implants where the TX and RX separation is of the order of several acoustic wavelengths, the PTE is sensitive to a change of the distance between TX and RX as well as temperature-dependent tissue properties and tissue growth. The misalignment of TX and RX can affect the PTE of APT drastically compared to EM-based WPT systems.
(3) The design of an APT transducer requires advanced design expertise than most of the EM-based WPT coils and antennas. Additionally, it is a costlier manufacturing process than EM WPT coils.

The tissue safety analysis is the primary research concern for the implementation of the APT systems for future IMDs. However, utilization of APT for vibration therapy can be an important future research topic [212].

### 7.6. Verdict

APT is the most popular non-EM WPT technique. It demonstrates promising potential in different IMD applications. The size of the RX of an APT is relatively small compared to NRIC and NRMRC. One of the concerning issues is the positioning of the RX transducers with respect to TX. The RX must be positioned within the radiation lobe of the TX to achieve higher PTE. Furthermore, analysis of tissue safety is still rarely presented for this WPT technique.

## 8. Optical Power Transfer

### 8.1. Link Design

**Figure 51:**
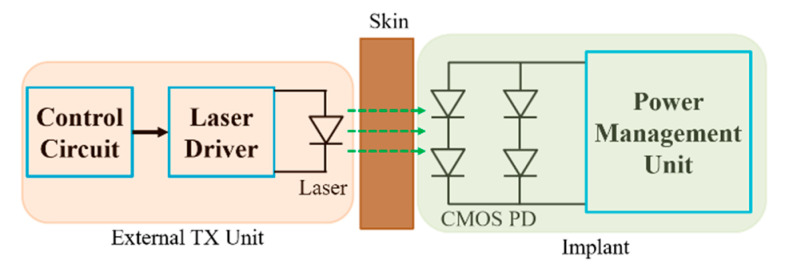
Complete optical power transfer (OPT) system.

Optical power transfer (OPT) is one of the least studied techniques to transfer power wirelessly to a biomedical implant [19,39]. Examples of OPT are rare [213,214,215,216] in the literature, even for other applications [213,214,215,216]. Figure 51 shows the complete OPT system where an external TX unit is used to power the implant utilizing an external laser. The light emitted by the external laser is received and converted to electric power by a CMOS photodiode (PD) array embedded under the skin using the power management unit. This method of WPT has the advantage of providing a compact solution as CMOS-based power receiver system can be smaller in size compared to other WPT systems [19].

### 8.2. Optimization

A PD-based RX system connected to a load can provide a small portion of its generated photocurrent, *J_sc_* to produce a nonzero load voltage, *V_load_*, such that [217]:

(47) $$V_{load}=\frac{nkT}{q}\ln\left[\frac{J_{sc}-J_{load}+1}{J_{O}}\right]$$

where *n*, *k*, *T* and *q* are the ideality factor, Boltzmann’s coefficient, absolute temperature and the absolute value of electron charge, respectively. *J_O_* is the reverse saturation current of the p-n junction and *J_load_* is the current through the load. In theory, the maximum available power is the product of maximum open-circuit voltage, *V_oc_* at the output and the maximum available short circuit current *J_sc_.* However, due to the loading effect it is not possible to reach maximum condition. Hence, a correction fill factor (FF) is defined as [217]:

(48) $$FF=\frac{J_{m}V_{m}}{J_{sc}V_{oc}}$$

where *J_m_* and *V_m_* are the output current density and the output voltage at the maximum power-point. The maximum cell output power using the FF is given as [217]:

(49) $$P_{m}=FFJ_{sc}V_{oc}$$

### 8.3. System Design

OPT systems for biomedical implantable devices are in very early stages of development compared to other methods, and the potential for this technology depends on the results of further study. Besides the design and manufacturing challenges, a lot more system implementation-based challenges have to be addressed before considering this system for sophisticated implants. A complete analysis of tissue safety is required for OPT. Additionally, misalignment and limited output power are significant concerns for the establishment of OPT for biomedical implant applications.

### 8.4. Applications

In [19], the maximum output power is claimed to be 168 μW for a 500 × 500 μm^2^ PD array. The achieved PTE inside 3-mm thick chicken skin is around 0.4%. Tissue safety is not analyzed in this work, and the actual medical application considered is not provided.

### 8.5. Verdict

OPT is the least studied WPT technique presently. The PTE is significantly lower to support most of the IMDs. Furthermore, any misalignment of the RX photodiodes with the TX can reduce the PTE drastically. Additionally, a rigorous tissue safety analysis is still required for this WPT technique before considering its usage in the IMDs.

## 9. Performance Comparison of Various WPT Schemes

**Figure 52:**
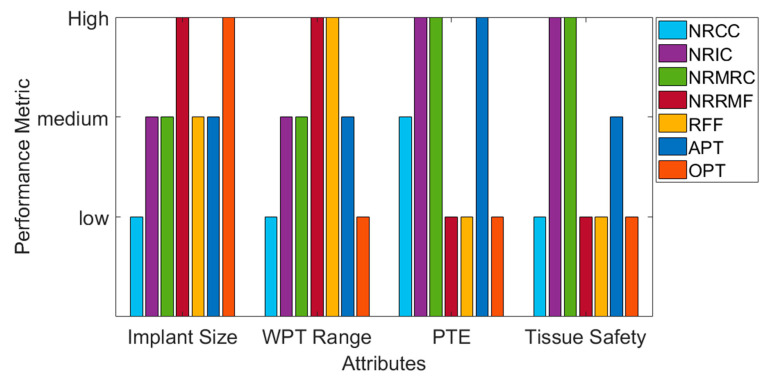
The abstract level figure of the performance of different WPT techniques.

IMDs are today being used for the diagnosis or treatment of pathologies ranging from brain, ocular, cochlear, heart to GI tract [111,218,219]. Given a specific IMD, various factors will affect the choice of a suitable WPT. These include implant RX size, range of power transmission, the volume of the RX electronic circuitry, PTE and tissue safety. A qualitative assessment of the performance—low, medium and high—of each of these factors is provided for each WPT scheme in Figure 52. NRIC and NRMRC outperform other WPT techniques due to their moderate size, range and higher PTE performance. Furthermore, unlike the other techniques, a comprehensive study on tissue safety exists in the literature for NRIC and NRMRC WPT schemes. APT is comparable to NRIC and NRMRC WPT in terms of performance. However, an extensive analysis of tissue safety is required before the increased utilization of this technique can occur. Most of the other methods (NRCC, NRRMF, RFF and OPT) are still in an early stage of research or development.

Figure 52 provides an indication of the suitability of a WPT technique for a given MID application. For example, an MID such as a brain implant requires a small size along with moderate WPT range (less than 100 mm). NRIC and NRMRC are the appropriate WPT techniques for this application due to their comparatively smaller size and the WPT link range of approximately 100 mm. It is also possible to achieve higher PTE by using these two techniques. However, it is necessary to consider the lower frequencies to avoid higher SAR. Therefore, one of the challenging factors is to achieve higher quality factor of implant coil to achieve satisfactory PTE.

Furthermore, NRIC and NRMRC are also appropriate techniques for applications such as neurostimulators, ocular and cochlear implants. Moderate implant RX coil size requirement of 10 to 20 mm can provide higher PTE in these applications using NRIC and NRMRC WPT techniques. The main design consideration for these implants is the tissue safety of the patients.

Capsule endoscopy requires free roaming of the RX coil in the patient’s GI tract with the requirement of power reception from any capsule direction. The radiative techniques NRRMF, RFF and APT provide more power focusing-based WPT approach compared to NRIC and NRMRC. In NRIC and NRMRC, the magnetic field is distributed evenly in the surrounding environment with the property of gradual decay with distance. However, with proper design of the TX coils in NRIC and NRMRC WPT, it is possible to supply sufficient power in capsule endoscopy application. It is necessary to fit the RX coil size in to a typical capsule size of 10 mm diameter and 25 mm in length. The RX coil must adopt 3D architecture to receive power from any direction. The selection of frequency for this application is largely depends on the tissue safety.

NRRMF and RFF WPT are suitable for the cardiac implants due to the deep implementation requirement. However, tissue safety must be maintained to design WPT systems using NRRMF and RFF techniques. APT can be another alternative for cardiac implant due to the nature of transmission of power at longer distances. APT can also be used for the applications such as photodynamic therapy by focusing the power in to a certain area inside the human body.

The manufacturing costs of NRCC, NRIC and NRMRC are lowest of all the techniques. These WPT techniques can be manufactured by using low cost copper wires or printing the coils in low cost materials such as FR4. NRRMF and RFF are comparatively costlier than NRIC and NRMRC as their implementation sometime requires special material and also careful design. APT and OPT are the most costly WPT techniques. These techniques require proper laboratory processing of wafer and costly manufacturing process.

Tissue safety of the patients is one of the most important factors to design WPT for MIDs. Tissue safety depends largely on how much EM field is absorbed in human body: it is a function of the microwave power density, frequency, absorption rate in the given tissue and the tissue sensitivity. At sufficiently low frequencies, the dielectric permittivity is constant and real-valued. Lower frequency waves penetrate deeper into the tissue, which offers little absorption at these frequencies, and, as there are fewer nerve endings located in the deep parts of the body, the effects of the radio frequency waves (and the damage caused) may not be immediately felt by the patient. As frequencies increase, the relative permittivity and conductivity of the human tissue decreases and increases, respectively, which increases the tissue absorption. Microwaves penetrate less into the body and tend to heat up the tissue more readily.

In wireless power transfer applications for MIDs, the primary measure of the tissue safety is considered as the specific absorption rate (SAR). It is the unit of measurement for the amount of radio frequency energy absorbed by a body when using a wireless device. SAR must be lower than the 2 W/kg for 10 g of tissue as per IEEE standard [60]. The industrial, scientific and medical (ISM) frequency bands are reserved internationally for WPT technology for MID applications. These bands include a lower kHz range (6.78, 13.56 and 27.12 kHz), lower MHz (6.78 MHz, 13.56 MHz and 27.12 MHz), higher MHz (433.9 and 915 MHz) and GHz (2.45, 5.8 and 24.125 GHz).

For NRIC WPT, the PTE is significantly dominated by the quality factor of the smaller size RX coils. Furthermore, the quality factor is function of frequency. With increase of frequency, the skin-effect in the coil increases significantly and the AC resistance increases, which reduces the current through the coil and the quality factor. Furthermore, at higher frequencies, the coils can also be moved towards their self-resonance frequency. Therefore, it is ideal to use lower kHz and MHz frequency bands for inductively coupled WPT techniques. However, the radiative far-field WPT works on the higher MHz and GHz frequency range. It must be noted that, at higher frequencies, the SAR can increase significantly. Therefore, the operation frequency of the WPT must be selected carefully for the efficient and safe operation of WPT for MIDs.

Furthermore, the following safety regulations are recommended with respect to static magnetic field strength, RF heating and time varying magnetic fields [35]:

(1) Typical exposure to static magnetic field must not exceed
  - 8 Tesla for adults, children and infants aged > 1 month.
  - 4 Tesla for infants aged ≤1 month.
(2) SAR for any WPT techniques should not exceed
  - 2 W/kg averaged over 10 g of tissue absorbing the most signal (Partial body SAR).
  - 4 W/kg averaged over whole body (Whole body SAR).
  - 3.2 W/kg averaged over head (Head SAR).
(3) Rate of change of magnetic field (B)
  - Any time rate of change of magnetic fields (dB/dt) should not cause discomfort or painful nerve stimulation.

Table 2 provides a more comparative and quantitative study, which summarizes the existing or researched IMDs for different WPT techniques presented in this work. Criteria considered include the size of the implanted RX, the separation distance between the TX and RX coils, frequency of operation, maximum achievable PTE, the measurement environment for different PTE and their tissue safety parameter for different IMDs.

Among all the WPT schemes, NRIC WPT is explored for a versatile type of IMDs. The maximum size of the implanted RX is 20 mm in diameter. This technique demonstrates higher PTE for smaller separation distance. Furthermore, the SAR is comparatively lower for higher input power; therefore, this technique is more acceptable for IMD applications currently.

NRMRC shows a higher PTE than NRIC with a smaller coil diameter. The tissue safety parameters are similar to the NRIC. NRRMF and RFF are smaller in size compared to NRIC and NRMRC. With higher separation distance and operation frequency, these techniques have lower PTE and higher SAR compared to NRIC and NRMRC.

Finally, the APT and OPT have a high potential for future WPT for IMDs with larger separation distance and smaller RX size. However, research on tissue safety parameters is still in infancy for these techniques.

It is worth mentioning that radio frequency identification (RFID) demonstrated significant potential for low and high frequency WPT and energy harvesting applications. However, its implementation using different WPT techniques can be a major challenge for MIDs [220,221].

## 10. Conclusions

This review article has presented various WPT platforms used for biomedical implantable devices and previously reported in the literature. Each WPT technique was broken down in terms of the presentation of the wireless mode of transmission (the link), the design and optimization methodology of the device, the design and implementation of the TX and RX circuits, the potential IMD applications that these schemes have been applied to and the different design challenges and the future research trends of each WPT technique.

The NRIC and NRMRC schemes have been investigated thoroughly over the last decade for IMDs and have become mature technologies for the FDA-approved cochlear and ocular implants. Device miniaturization and engineering of flexible interfaces are still under development for implants that need to be placed in critically curved locations in the human body, such as the heart and brain.

The NRCC WPT scheme is facilitated by the use of flexible patches, but the PTE drops drastically with increasing TX and RX separation distance. Tissue safety still needs further research for this technique.

NRRMF and RFF WPT techniques can transfer power deep inside the human body. However, these powering schemes suffer from low PTE, which limits the applications to those where ultra-low-power electronics are permitted inside the IMD. Furthermore, the vulnerability of tissue due to higher radiation frequency is a challenging issue for these platforms. APT uses ultrasonic waves to transfer energy through a medium to the IMD. This technique is limited by significant PTE variation due to the misalignment and long-term effects of the tissue vibration that is yet to be studied to ensure its safety for in vivo testing. OPT is the least studied WPT technique, but this technique could potentially reduce the size of the RX significantly. This technique requires substantial research to evolve to its potential usability.

In summary, NRIC and NRMRC are still considered as the most suitable WPT strategies to meet the power requirements of IMDs while maintaining the health of the surrounding tissue. However, this review work opens a possibility for experienced researchers to compare different WPT techniques and focus on overcoming the shortcomings of the specific WPT scheme targeting IMD applications. This body of work also suggests future alternative research opportunities of different WPT techniques targeted toward applications, such as patient treatment and localization of IMD. Moreover, it is hoped that this review article will assist new researchers in identifying the domains of interest for future developments in the field of WPT for IMD applications.

## Figures and Tables

**Table 1:** Current sheet expression coefficients.

Layout	*C*_1_	*C*_2_	*C*_3_	*C*_4_
Circle	1.27	2.07	0.18	0.13
Octagonal	1.09	2.23	0.00	0.17
Hexagonal	1.07	2.29	0.00	0.19
Square	1.00	2.46	0.00	0.20

**Table 2:** A comparison of different WPT techniques for IMD applications includes power budget, PTE and SAR to allow the reader decide particular technique of choice.

Powering Scheme	Year/Ref	Implant Type	Implant WPTRX Size	Distance (mm)	Frequency (MHz)	Input Power (W)	PTE (%)	Test Model	SAR (W/kg)	Remarks	Maturity for IMD
NRCC	2017/[53]	Generic	20 mm × 20 mm	7	100–150	1	56	non-human primate cadaver	8.02	TX and RX are large. Separation distance and tissue safety low.	Low
NRIC	2018/[40]	Brain	Diameter: 4 mm (around-CMOS)	11	318.8	0.01	3.05	Lamb ribs	0.155	Acceptable PTE for medium separation distance. Wide range of application. Tissue safety is achieved. The circuit for TX and RX is easily implementable and cheap.	High(implemented in some commercial IMDs)
	2017/[106]	Brain	Volume: 0.9 mm^3^, Gap: 0.1 mm	3	402	0.082	0.08	Piglet	1.97		
	2015/[111]	Peripheral Nerve	20	5	1	0.18	65.8	Rat stomach	0.1		
	2017/[112]	Ocular	Diameter: 20 mm	8 (Max. 40 mm distance)	2	-	60 (5% PTE for 40 mm)	Beef muscle	0.66		
	2015/[113]	Ocular	Diameter: 10 mm	20	13.56	2	17.5	Pig eye	0.021		
	2007/[99]	Capsule	10 mm × 13 mm	-	1	-	1	Air	0.32		
	2010/[122]	Capsule	10 mm × 12 mm	-	0.4	-	1.2	Air	0.329		
	2011/[121]	Capsule	11.5 mm × 11.5 mm	200	0.218	-	5.5	Air	8		
	2012/[103]	Capsule	Diameter:11 mm	-	13.56	8	3.04	Phantom	0.1		
NRMRC	2017/[145]	Brain	Diameter: 1.2 mm	16	60	-	3	Fresh lamb head	Less than 1.6	RX size is smaller than NRIC and the separation distance is higher. Tissue safety is within the limit. Better impedance matching than NRIC.	High (possible to implement in commercial IMDs)
	2012/[146]	Ocular	Diameter: 15 mm	10	3.37	-	62.5	Air	-		
	2011/[147]	Ocular	Diameter: 15 mm	5	6.78	-	8.8	Human head	-		
	2015/[148]	Capsule	Diameter: 9 mm	70	16.47	150	0.7	Chopped pig tissue	1.74		
	2016/[149]	Capsule	15 mm × 7 mm × 6 mm	50	433.9	1	1.21	Duck intestine	2.54		
NRRMF	2014/[156]	Cardiac	Diameter: 2 mm	50	1600	0.5	0.04	Epicardium of rabbit	0.89	Higher separation distance for smaller RX. PTE is low. Tissue safety is alarming.	Medium (more research required for IMD)
	2017/[160]	Peripheral Nerve	20 mm	15	2400	0.18	20	Right neck of pig	2		
	2017/[161]	Capsule	Printed on capsule	-	402–405	-	0.08	A phantom containing a porcine heart	-		
RFF	2011/[183]	Generic	10 mm × 10 mm × 2 mm	-	433	0.005	15	Minced pork of 65 mm × 92 mm × 50 mm	1.6	Higher distance. Lower PTE and tissue safety is alarming.	Medium
	2010/[181]	Cardiac	-	100	3700	3.2	-	Chest cavity of porcine	2.2898		
APT	2011/[206]	Micro-oxygen generator	5 mm^2^	30 mm	2.15	-	-	Pancreatic tumor of athymic mice	-	Promising WPT technique with smaller RX and large separation distance compared to NRIC and NRMRC. Tissue safety study is necessary.	High (more research required to implement in commercial IMDs)
	2014/[203]	Bladder pressure	20 mm × 2 mm × 0.38 mm	100	0.00035	-	1.4 × 10^−4^	Pig bladder	-		
	2015/[207]	Photo dynamic therapy	2 mm × 2 mm × 2 mm	10	0.672	-	-	Porcine tissue	-		
	2018/[205]	Peripheral nerves	-	105	1.314	-	-	Frog static nerve	-		
OPT	2015/[19]	Generic	0.5 mm × 0.5 mm	3	-	-	0.4	Chicken skin	-	Early stage in research.	Low

Note: Empty spaces indicates that the information is not available.
